# NSAID use and somatic exomic mutations in Barrett’s esophagus

**DOI:** 10.1186/s13073-018-0520-y

**Published:** 2018-02-27

**Authors:** Patricia C. Galipeau, Kenji M. Oman, Thomas G. Paulson, Carissa A. Sanchez, Qing Zhang, Jerry A. Marty, Jeffrey J. Delrow, Mary K. Kuhner, Thomas L. Vaughan, Brian J. Reid, Xiaohong Li

**Affiliations:** 1 Division of Human Biology, Fred Hutchinson Cancer Research Center, PO Box 19024, 1100 Fairview Ave N, Seattle, WA 98109-1024 USA; 2 Bioinformatics Shared Resource, Fred Hutchinson Cancer Research Center, PO Box 19024, Seattle, WA 98109-1024 USA; 3 Genomics Shared Resource, Fred Hutchinson Cancer Research Center, PO Box 19024, Seattle, WA 98109-1024 USA; 4 Genomics and Bioinformatics Shared Resources, Fred Hutchinson Cancer Research Center, PO Box 19024, Seattle, WA 98109-1024 USA; 5 Department of Genome Sciences, University of Washington, Foege Building S-250, Box 355065, 3720 15th Ave NE, Seattle, WA 98195-5065 USA; 6 Department of Epidemiology, University of Washington, Division of Public Health Sciences, Fred Hutchinson Cancer Research Center, PO Box 19024, Seattle, WA 98109-1024 USA; 7 Department of Medicine, University of Washington, Division of Human Biology, Fred Hutchinson Cancer Research Center, PO Box 19024, Seattle, WA 98109-1024 USA

**Keywords:** Exome sequencing, Mutation, Apoptosis, Barrett’s esophagus, Esophageal adenocarcinoma, Aspirin, NSAID, Tobacco smoking, Cancer prevention, *TP53*

## Abstract

**Background:**

Use of aspirin and other non-steroidal anti-inflammatory drugs (NSAIDs) has been shown to protect against tetraploidy, aneuploidy, and chromosomal alterations in the metaplastic condition Barrett’s esophagus (BE) and to lower the incidence and mortality of esophageal adenocarcinoma (EA). The esophagus is exposed to both intrinsic and extrinsic mutagens resulting from gastric reflux, chronic inflammation, and exposure to environmental carcinogens such as those found in cigarettes. Here we test the hypothesis that NSAID use inhibits accumulation of point mutations/indels during somatic genomic evolution in BE.

**Methods:**

Whole exome sequences were generated from 82 purified epithelial biopsies and paired blood samples from a cross-sectional study of 41 NSAID users and 41 non-users matched by sex, age, smoking, and continuous time using or not using NSAIDs.

**Results:**

NSAID use reduced overall frequency of point mutations across the spectrum of mutation types, lowered the frequency of mutations even when adjusted for both *TP53* mutation and smoking status, and decreased the prevalence of clones with high variant allele frequency. Never smokers who consistently used NSAIDs had fewer point mutations in signature 17, which is commonly found in EA. NSAID users had, on average, a 50% reduction in functional gene mutations in nine cancer-associated pathways and also had less diversity in pathway mutational burden compared to non-users.

**Conclusions:**

These results indicate NSAID use functions to limit overall mutations on which selection can act and supports a model in which specific mutant cell populations survive or expand better in the absence of NSAIDs.

**Electronic supplementary material:**

The online version of this article (10.1186/s13073-018-0520-y) contains supplementary material, which is available to authorized users.

## Background

Comprehensive analysis of cancer genomes has firmly established that natural selection acts on the diversity of somatic mutations and chromosomal alterations generated through genomic instability to promote neoplastic evolution of cancer [1]. In esophageal adenocarcinoma (EA), this process can be accelerated by both instrinsic mutagenic exposures, such as oxidative stress and genotoxicity induced by chronic inflammation [2], and extrinsic mutagens, such as acid, bile, and carcinogens found in tobacco [3–6]. In randomized trials for other indications, aspirin and other non-steroidal anti-inflammatory drugs (NSAIDs) have been reported to be effective chemopreventive agents for many cancer types, including EA [7–9]. However, the effects of NSAID use on somatic mutations such as single nucleotide variants (SNVs) and small insertions/deletions (indels) and how NSAID use protects against cancer incidence and mortality are not well understood.

EA is characterized by frequent *TP53* mutations, a high frequency of somatic point mutations across the genome, extensive somatic chromosomal alterations (SCA), whole genome doubling (WGD), aneuploidy, and complex structural rearrangements such as chromothripsis [5, 10–17]. Barrett’s esophagus (BE), the precursor to EA, is a metaplastic condition in which the normal squamous epithelium is replaced by a crypt-structured columnar epithelium that has been proposed to function as a protective adaptation to the damaging reflux environment (reviewed in [18, 19]). Use of aspirin and other NSAIDs in BE patients has been reported to reduce risk of DNA content tetraploidy, aneuploidy, and progression to EA [20–23]. Additionally, NSAIDs have been shown to reduce the rate at which chromosomal alterations accumulate over time in BE [24]. These findings suggest that interventions with NSAIDs function in part to reduce the frequency of chromosomal alterations induced by the genotoxic environment of the reflux-exposed esophagus. Barrett’s epithelium is a chronically inflamed tissue [2] which creates a complex environment of oxidative stress and genotoxicity and which also selects for defective, decreased, or dysregulated DNA repair, cell cycle checkpoints, and apoptosis pathways that underlie genomic instability and evolution to cancer [25, 26]. Large-scale chromosomal alterations, aneuploidy, and WGD are more common in individuals with BE who progress to EA compared to non-progressors and can be detected two to four years before EA diagnosis [14, 27]. Conversely, with the exception of a small number of genes including *TP53*, genes mutated in EA are typically detected at similar frequency in BE adjacent to EA [10, 12–14, 16]. To our knowledge, there have been no well-designed studies of the influence of NSAID use on somatic SNVs and indels in BE.

Recent approaches have been developed to decipher mutational signatures arising from different mutagenic processes leading to the spectrum of mutations detected in a cancer [28–34]. Both BE and EA are characterized by two predominant mutation signatures [5, 10–14, 16, 32, 35]. Signature S1 is initiated by spontaneous deamination of 5-methylcytosine which accumulates over cell divisions in a clock-like manner, is associated with aging, and is common across many cancer types. Signature S17 is characterized primarily by T > G and T > C substitutions at CTT trinucleotides and has been consistently reported in EA and gastric cancers. Large-scale studies describing the EA genomic landscape have characterized snapshots of mutational profiles in advanced cancers and surrounding BE tissue, but critical information relative to whether NSAID use alters mutation signature patterns is missing because NSAID use status was unknown and/or control populations that did not progress to cancer were not available for comparison.

In this study, we hypothesized that mutant cells survive and/or expand better in the absence of NSAIDs. A cross-sectional study was designed from a well-annotated cohort of individuals with BE enrolled in a periodic endoscopic EA surveillance program in which data on medication and cigarette use were also collected [36, 37]. Whole exome sequencing (WES) was used to test the modulating effect of NSAID use on exomic mutations in a premalignant condition in human tissue in vivo. Overall somatic mutations in their trinucleotide context, variant allele frequency (VAF), mutatagenic signatures, and diversity of gene pathways altered by SNVs and indels were compared in individuals with BE after a period of continuous NSAID use compared to those who did not use NSAIDs.

## Methods

For each section below, see Additional file 1 for a complete description of the materials and methods used to generate this dataset and results.

### Study design

Participants were selected from The Seattle Barrett’s Esophagus Study using a cross-sectional study designed to take into account NSAID use/non-use, time on or off of NSAIDS, sex, age, and smoking status, resulting in 41 NSAID users and 41 non-users.

### Sample processing, library preparation, sequencing, alignment, and mutation calling

DNA was extracted from epithelial-enriched endoscopic biopsies from the middle of the BE segment. Pre-capture KAPA sequencing libraries were prepared and sequences with NimbleGen SeqCap EZ Exome + UTR library set and sequenced on an Illumina HiSeq 2500 using a paired-end 100-base read depth. Paired-end reads were aligned using BWA, v0.7.10 [38] and variants called using MuTect [39] and Strelka [40].

### Frequency analysis of point mutation sites in their tri-nucleotide context and overall mutation load

SNVs were compared between NSAID users and non-users using a sign test on the differences in median values across the 96 tri-nucleotides and a Kruskal–Wallis test to compare a difference in total mutation load (SNV and indel).

### Mutation annotation for gene functional impact

Each SNV and indel was annotated for functional impact on RefSeq transcripts [41].

### Additional statistical tests for overall mutation load

A trend test to compare the mutation load (combined count of SNVs and indels) was employed to compare the difference in load across functional groups for NSAID users vs non-users. Additionally, a mixed effects model was used to compare mutation density (mutation load per chromosome arm divided by arm length) between NSAID users and non-users.

### *TP53* mutation and NSAID effect on mutation load

For samples with a *TP53* mutation, mutation load (total SNVs and indels per patient) was compared between NSAID users and non-users with a Kruskal–Wallis test, as well as with a sign test across the 96 tri-nucleotide context of SNVs. Additionally, a multivariable regression model tested the effect of NSAID use, smoking status, and *TP53* mutation status on total mutations and total functional mutations.

### Variant allele frequency comparison

Variant allele frequency (VAF) was compared for both total and functional SNVs to determine a VAF threshold with a significantly different number of SNVs between NSAID users and non-users.

### Mutation signature discovery

Mutation signature contributions to the mutational profile were determined by both the non-negative matrix factorization method developed by Alexandrov et al. [42], as well as using deconstructSigs [32].

### Mutation signature analysis

Linear regression models (single or multivariable) were used to evaluate the significance of correlation between parameters of interest (NSAID use, smoking status, etc., as binary variables) to the SNV mutation load contributions from each signature. For the deconstructSigs analysis, six individuals (four NSAID users and two non-users) were assigned values of 0 mutations from all signatures because they had fewer than the minimum 50 recommended SNVs [32]. As the distribution of number of mutations per patient attributed to each signature (including from the aforementioned six individuals) tended to be right-skewed, one was added to the number of mutations for that signature per patient (to include those participants that did not have any mutations attributed to a signature, in deconstructSigs analysis only, as Alexandrov always has mutations associated to both S1 and S17 for each sample), then log transformed these values.

### Gene-by-gene mutation association and analysis

Total functional mutations (see definition in the “Mutation annotation for gene functional impact” section) per individual were determined and NSAID users were compared to non-users using a Kruskal–Wallis test, with additional thresholds applied first, then *p* values adjusted to control the false discovery rate (FDR) using the Benjamini–Hochberg procedure [43] with FDR = 0.1 or 0.2. See Additional file 1 for details.

### Pathway selection/processing

Thirty-five pathways of interest were obtained from the Network Data Exchange [44, 45] downloaded on 26 September 2017 (Additional file 2: Table S1), and the regulatory pathway for COX-2 expression gene list was generated from Kang et al. [46].

### Mutation pathway assignment and analysis

SNVs and indels within 1 kb of a gene were assigned to pathways containing that gene. Total number of functional mutations in each pathway (see definition in the “Mutation annotation for gene functional impact” section) was determined for each patient, with a Kruskal–Wallis test performed per pathway of these mutation counts comparing NSAID users vs non-users, with the Benjamini–Hochberg procedure [43] employed to adjust the *p* value threshold to control the FDR = 0.2. An analogous test was also performed for non-functional mutations.

### Pathway mutation diversity assessment

Pathway mutation diversity quantifies the number of pathways affected by point mutations and indels. The Shannon index was modified to assess this diversity and a Kruskal–Wallis test was used to compare differences in diversity between NSAID users and non-users.

### Somatic chromosome copy number and LOH segmentation

Twelve DNA samples in this study had been run on 1 M Illumina SNP arrays in a previous study [27]. SNP arrays are a robust platform for SCA measurements [47] and were used as a point of comparison in the SCA calls made using the exome data of this study (See Additional file 1 for details).

### Somatic chromosome alterations analysis

A mixed effects model was utilized to assess the effect of NSAID use on the SCA load (MB of SCA) and the number of SCA segments (see Additional file 1).

## Results

### Overall exomic mutations

**Fig. 1:**
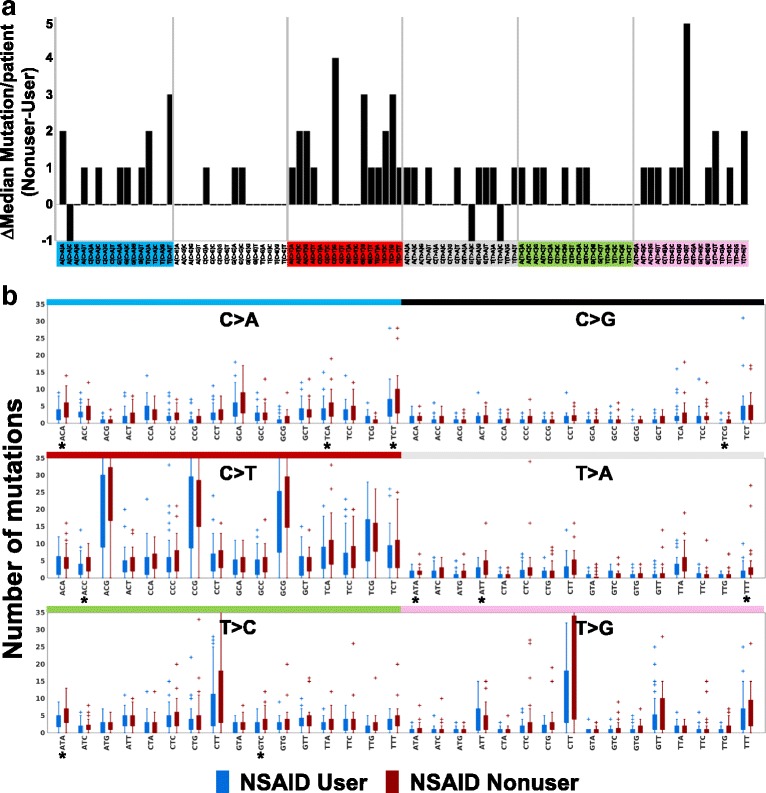
Differences in 96-trinucleotide somatic base substitutions between NSAID users and non-users. **a** Difference in median mutation load at each 96 trinucleotide somatic base substitution between NSAID non-users and users. Medians above zero are higher in NSAID non-users. **b** *Box plot* for mutation levels in NSAID user (*blue*) and NSAID non-user (*red*) at each 96 trinucleotide somatic base substitution. Ten substitutions (indicated by *) had significantly different median mutation level including the following (mean mutation count in user/non-user, *p* value): C > A at ACA (2.78/4.37, *p* = 0.03) TCA (3.2/4.59, *p* = 0.02), and TCT (5.1/7.1, *p* = 0.04); C > G at TCG (0.22/0.59, *p* = 0.003); C > T at ACC (2.98/4.17, *p* = 0.0099); T > A at ATA (0.90/1.58, *p* = 0.01), ATT (1.63/3.05, *p* = 0.02) and TTT (1.51/3.2, *p* = 0.015); T > C at ATA (3.12/4.61, *p* = 0.008) and GTC (2.0/3.24, *p* = 0.02), Kruskal–Wallis

To test whether NSAID users have a lower somatic mutation load compared to non-users, WES was performed in samples from blood and paired epithelium purified from one BE biopsy per individual from the middle of the BE segment taken at the end of a period of consistent NSAID use in 41 NSAID users compared to 41 matched non-users (Additional file 2: Table S2, Additional file 3: Figure S1). Participants were defined as NSAID users if they consistently reported at each interview during the assessed time-period that they had been using aspirin or other NSAIDs at least once a week for six months or more. A total of 28,430 somatic SNV mutations were independently called by two variant calling algorithms, with a mean of 290.93 (range = 13–949, SD = 209.69) and 402.49 (range = 7–1831, SD = 362.68) SNVs/biopsy in NSAID users and non-users, respectively (Additional file 2: Tables S3–S5). Somatic SNVs were classified according to the six substitutions referred to by the pyrimidine of the mutated Watson-Crick base pair in the context of 5′ and 3′ bases, giving rise to 96 somatic base substitutions in their trinucleotide context (Fig. 1; Additional file 3: Figure S2). Of the 96 base substitutions, 46 had a lower median mutation count in users compared to non-users, while only three of the 96 had higher median mutation count in NSAID users. The difference in median mutation load between NSAID users compared to non-users across the 96 substitutions in their trinucleotide context was statistically significant (sign test, *p* < 3 × 10^−16^).

A total of 1655 indels were detected, with a mean of 17.95 (range = 1–50, SD = 12.25) and 22.42 (range = 1–75, SD = 13.77) indels detected in NSAID users and non-users, respectively (Additional file 2: Tables S6 and S7). NSAID users had a lower median combined mutation load (SNVs and indels) with 2.95 mut/Mb (range = 0.15–10.28 mut/Mb) compared to 3.46 mut/Mb (range = 0.08–19.55 mut/Mb) for non-users, but the overall difference in median total mutation load per biopsy between users and non-users did not reach statistical significance (*p* = 0.104, Kruskal–Wallis). These mutation frequencies are within the range of the median exome SNV/indel mutation frequency reported for non-dysplastic (2.8 mut/Mb) and dysplastic BE adjacent to EA (4.9 mut/Mb) and EA (4.1 mut/Mb) [14]. Across 15 mutation categories (see Additional file 1), there was a highly significant trend for users to have fewer mutations (SNVs and indels) than non-users (sign test *p* < 9.8 × 10^−04^; Additional file 2: Tables S5 and S7). This significant difference is also seen when testing mutation load across the genome segregated by chromosome arm (Additional file 1) showing significantly fewer mutations in users compared to non-users for non-functional mutations (*p* = 0.04) and marginally fewer for functional mutations defined as exon-non-synonymous, 3’utr-exon, 5’utr-exon, coding-splicing, and utr-splicing (*p* = 0.077), evoking a mechanism by which NSAID use either reduces overall mutagenic potential in the tissue environment or inhibits expansion of cells with more mutations.

*TP53* plays an essential role in maintaining genomic stability and has been consistently shown to be the most frequently mutated gene in EA. Out of the 82 biopsies in the study, a total of 19 functional *TP53* point mutations were detected in 6/41 NSAID users and 11/41 NSAID non-users, with two non-users having two different *TP53* mutations/biopsy (Additional file 2: Table S8). Given that mutant p53 is predictive of progression to EA [21, 48, 49], we evaluated the effect of NSAID use on overall mutations in participants with and without mutant *TP53*. Individuals with *TP53* mutation had a significantly higher number of mutations (SNV and indels) than those with wild-type (WT) *TP53* (*p* = 1.3 × 10^−4^, Kruskal–Wallis). Although the number of individuals with *TP53* mutation was small, within the 17 participants with mutant *TP53*, there was a significant difference in median point mutation frequency across the 96 possible mutations in their trinucleotide context comparing NSAID users and non-users (*p* = 0.01, sign test). In the larger set of individuals with WT *TP53* (*n* = 35 users, 30 non-users), the difference in median distribution of point mutations across the 96 possible mutations between NSAID users and non-users was highly significant (*p* < 3 × 10^−9^, sign test). Smoking is known to increase mutation frequency [6]. Therefore, both *TP53* mutation status and smoking status were taken into account with a multivariable regression analysis. This analysis showed NSAID users had significantly lower total SNV and indel mutations (*p* = 0.026) and total mutations with likely functional effects in coding regions (*p* = 0.021, Additional file 1).

### Variant allele frequency

One hypothesis is that NSAID use prevents the expansion of cell populations with genomic alterations [24], possibly through suppression of inflammation and reduced cell turnover. To test this hypothesis, point mutation variant allele frequency (VAF) was used as a measure of clonality and compared between users and non-users [50]. Only mutations from diploid regions with no copy number or copy neutral loss of heterozygosity (cnLOH) alterations were evaluated to avoid the confounding effect of copy number loss, gain, and cnLOH on VAF [51]. The VAF distribution pattern in diploid regions was compared between NSAID users and non-users for functional and non-functional point mutations separately. NSAID users had significantly fewer functional point mutations with VAF > 0.3 compared to non-users (*p* = 0.013), as well as for non-functional point mutations (*p* = 0.006; Additional file 1). Similar results were obtained when controlling for total number of mutations per sample (functional mutations *p* = 0.06, non-functional mutations *p* = 0.025). Comparably, a previous report proposed a definition of predominant clones as those with mutations with VAF > 0.25 [52], suggesting NSAID users have significantly fewer mutations from predominant clones than non-users. These data suggest NSAID use not only lowers overall mutation load but also functions to keep mutant cell populations from expanding to become predominant clones in the BE epithelium.

### Mutagenic signatures

**Fig. 2:**
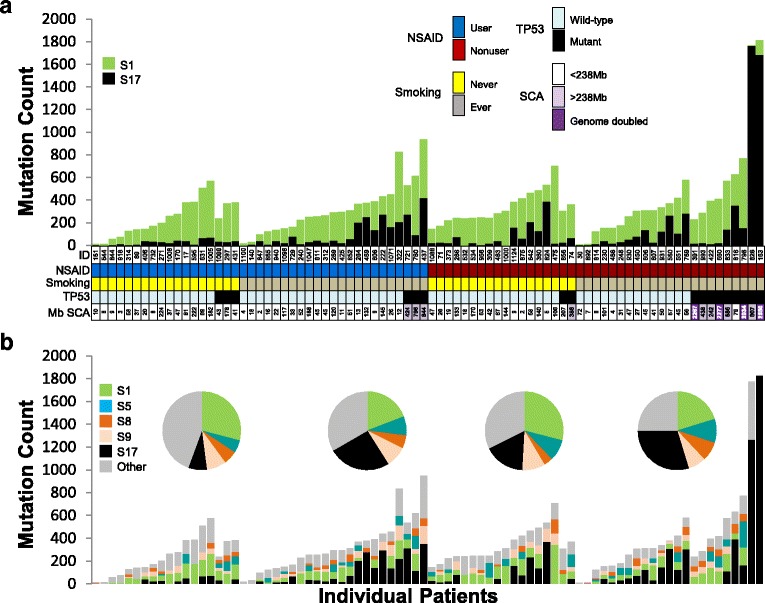
Mutation signatures in NSAID users and non-users. **a** Deconvoluting the mutation profile across the cohort into stable signatures using the method of Alexandrov et al. [29] yields a two-signature solution comprising COSMIC S1 and S17. **b** DeconstructSigs, an independent method of determining COSMIC mutation signature contributions to per-individual mutation profiles, shows COSMIC S1 and S17 as dominant, with relative proportions sensitive to NSAID use and smoking status. The relative contribution of the top five signatures (which had ≥ 5% of the total mutations associated with them) to each patient category (*pie charts*) and to each patient (*bar charts*) is shown

Exposure to exogenous and endogenous DNA damage over an individual’s lifetime [33], and of the functional state of DNA damage sensing and repair mechanisms, each contribute to the point mutation composition in the Barrett’s epithelium. To test the hypothesis that NSAID use modifies the signature of point mutations based on their flanking nucleotide context, the 96 possible mutation configuration in the three-base contexts were assessed across all participants using the non-negative matrix factorization (NMF) method developed by Alexandrov et al. [29] and using deconstructSigs to determine the optimal mixture of pre-defined signatures that best fits the observed mutational profile in each individual [32]. Using the NMF method, two high-confidence signatures were identified, consistent with COSMIC Consensus Signatures 17 and 1 [34] (Fig. 2a, Additional file 2: Table S9; Additional file 3: Figure S3). Signature 17 is characterized by T > G transversion mutations and T > C transitions at CTT sites, whereas Signature 1 is common in all epithelial cancers and is enriched in C > T transition mutations, likely reflecting spontaneous deamination of methylated cytosines. Using deconstructSigs, which infers weighted contributions of mutations from each predefined signature for each individual sample, the signatures contributing > 5% across all individuals were S17 (31.5%), S1 (20.5%), S9 (7.8%), S5 (7.1%), and S8 (5.5%) (Fig. 2b*,* Additional file 2: Table S10).

When NSAID use was evaluated alone, neither S17 nor S1 mutations were enriched in NSAID non-users compared to users (Alexandrov method: *p* = 0.53, 0.33, respectively, deconstructSigs: *p* = 0.63, 0.20, respectively), suggesting NSAID lowering of mutation load is not unique to a particular point mutation context. When smoking status was evaluated alone, ever smokers were enriched for S17 mutations compared to never smokers (Alexandrov method: *p* = 0.011, deconstructSigs: *p* = 0.011), but no significant difference was seen for S1 mutations, signifying an interaction between smoking and the other mutational processes present in BE that results in an increase of S17 mutations. However, when NSAID users who never smoked were compared to the rest of the study participants, they had significantly fewer S17 mutations compared to all other participants (Alexandrov method: *p* = 0.014, deconstructSigs: *p* = 0.018). In contrast, no difference was found for these comparisons for S1, S5, S8, or S9 mutations using either method.

In the 15 samples with mutant *TP53* with VAF > 0.3, *TP53* mutant samples had a higher number of S17 mutations than WT *TP53* samples in both NSAID users (*p* = 0.04) and non-users (deconstructSigs: *p* = 0.026, single variable linear regression model), but no difference was seen for S1. When *TP53* mutations, NSAID use, and smoking status were considered together, only smoking remained significant for the number of S17 mutations (deconstructSigs: *p* = 0.015).

### Functional mutation loads in gene pathways

Consistent with previous sequencing studies in both BE and EA, only a small number of genes with likely functional somatic SNV or indel mutations were detected in more than two participants, reducing the power to efficiently test the NSAID effect on mutations at the single gene level. Despite this limitation, 31 genes had a significantly different functional SNV/indel mutation load between NSAID users and non-users (using thresholds ≥ 5 users or non-users with functional gene mutation, FDR = 0.1; Additional file 2: Table S11). NSAID users had fewer functional mutations than non-users in 29/31 of these genes, whereas only two genes had more functional mutations in NSAID users. A total of 1125 genes had significantly different functional mutation load between NSAID users and non-users using a relaxed threshold.

**Fig. 3:**
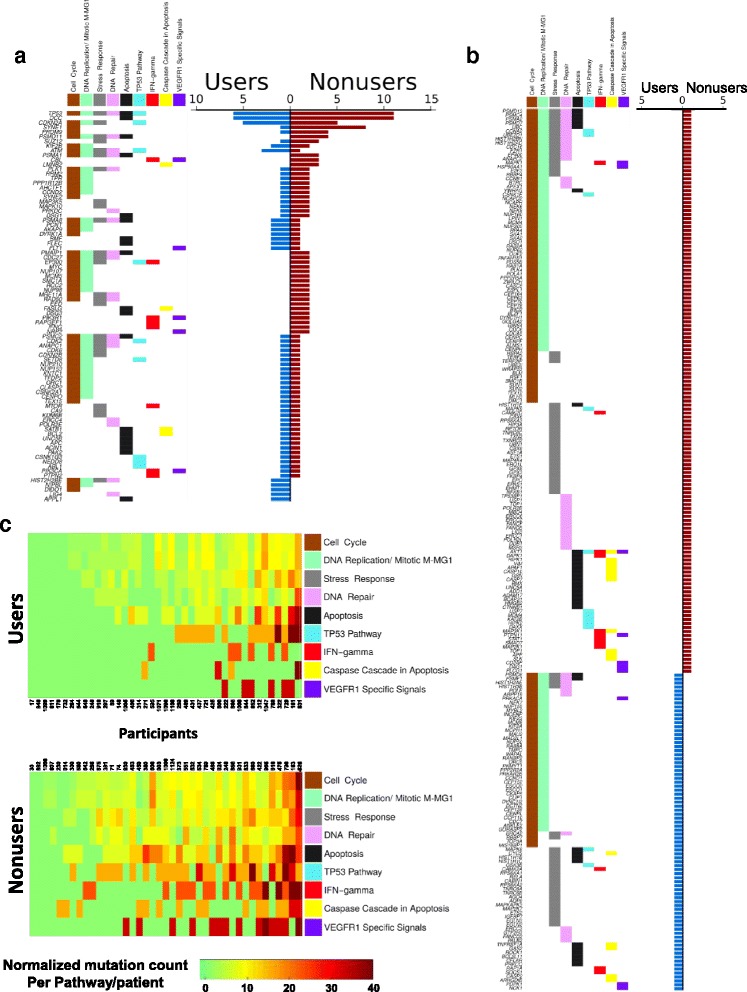
Pathway mutations and diversity. **a** Pathways with significantly lower functional SNV/indel mutations in NSAID users vs non-users are plotted in *columns*, with pathways ordered left to right by total number of genes with functional mutations in each pathway. For each gene, the *left plot* displays a *colored box* in one or more pathways in which that gene is classified. Genes names are ordered from top to bottom by number of participants with at least one functional mutation in that gene, then by NSAID non-users, then users. The *right plot* shows the count of NSAID users (*blue*) and non-users (*red*) with at least one functional mutations in each gene. **b** Same plot organization as in (**a**) for genes with functional mutations in only NSAID user (*blue*) or in only non-user (*red*). Most genes had functional mutations in only one individual, highlighting the heterogeneity of mutated genes across participants. **c** NSAID use selects against diversity of mutations across pathways. The number of functional mutations per participant, per pathway, normalized by the number of genes per pathway, is shown for users (*top heatmap*) and non-users (*bottom heat map*)

Given the heterogeneity of mutated genes across patients, a pathway analysis was used to test if NSAIDs select against SNV and indel mutations in specific pathways important for neoplastic evolution to cancer. We examined 35 pathway gene lists of interest (Additional file 2: Table S11). For each pathway, both likely functional and presumed non-functional SNV/indel mutation load was compared between NSAID users and non-users (Additional file 2: Table S12). NSAID users had significantly fewer functional mutations in nine pathways (Table 1, Fig. 3a and b, Additional file 2: Table S12). In contrast, with the exception of the *TP53* pathway, in 8/9 of these pathways there was no significant difference in non-functional mutations, indicating there is specific selection against functional mutations in these pathways in individuals using NSAIDs, beyond the more general lowering of mutations overall (Additional file 2: Table S11). NSAID use reduced functional mutations in each of the nine pathways by 37–74% compared to non-users. These nine pathways comprised 906 unique genes (Additional file 1), with 563/906 genes having at least one somatic SNV/indel mutation (regardless of function) in this study. Across all BE study participants, eight genes in these pathways had functional mutations in ≥ 5% of participants, including *TP53* (21%), *DCC* (21%), *CDKN2A* (12%), *SYNE1* (11%), *PRDM9* (6%), and *ATM*, *KIF2B*, *PSMD11* (each at 5%) (Additional file 2: Table S13), but most genes were mutated in only a single individual.

**Table 1:** Pathways with significantly lower gene mutations in NSAID users

Pathway	Functional mutations in pathway per NSAID user (mean (SEM))	Functional mutations in pathway per NSAID non-user (mean (SEM))	Mutation reduction in NSAID users (%)	*p* value
DNA repair	0.83 (0.18)	1.71 (0.24)	− 51.43	0.003
Apoptosis	1.00 (0.23)	1.76 (0.25)	− 43.06	0.004
Caspase cascade in apoptosis	0.17 (0.09)	0.44 (0.09)	− 61.11	0.004
IFN-gamma pathway	0.15 (0.06)	0.56 (0.13)	− 73.91	0.005
Cellular response to stress	1.41 (0.25)	2.46 (0.33)	− 42.57	0.009
Cell cycle	2.39 (0.33)	4.20 (0.64)	− 43.02	0.014
p53 pathway	0.51 (0.13)	0.88 (0.15)	− 41.67	0.019
VEGFR1 specific signals	0.15 (0.06)	0.39 (0.09)	− 62.5	0.035
DNA replication/Mitotic M-MG1 phases	2.12 (0.31)	3.37 (0.48)	− 36.96	0.042

### Diversity of pathway mutations

An application of the Shannon index (SI) was used to assess the distribution of genes with likely functional SNV/indel mutations across the nine significantly altered pathways (Fig. 3c, Additional file 1, Additional file 2: Table S14). This modified SI quantifies how the mutations are distributed across the nine pathways within each patient (Additional file 1). NSAID users had significantly lower SI (lower diversity of genes with functional mutations across the nine pathways) than non-users across these nine pathways (*p* = 0.007). A similar test across all combined remaining 25 pathways also showed lower diversity of functional pathway mutations in NSAID users, but the result was not significant (*p* = 0.16). Higher pathway mutation diversity (high SI) indicates mutations were more abundant and/or more highly distributed across the nine pathways. For example, a patient having mutations distributed across all nine pathways will have a higher SI than another patient having the same number of mutations all occurring in a single pathway. Thus, NSAID use substantially reduced diversity of functional mutation frequency across these nine pathways, thus reducing mutations on which selection can act during neoplastic evolution.

### Chromosomal copy number alterations and LOH

Previous studies in BE using DNA content flow cytometry, which measures tetraploidy and aneuploidy directly, have shown that NSAID use may act to eliminate or prevent development of cells that have undergone WGD [21]. Studies using SNP arrays, which allow for inference of a previous WGD event, have shown that NSAID use slowed or reduced the accumulation of somatic chromosomal alterations (SCA) [24]. In our study, SCA was quantified from exome data using an expanded algorithm based on a modified version of ADTEx (Additional file 2: Table S15) and binned into SCA classes including homozygous deletions (HD), copy loss, cnLOH, balanced gain (> 2 N copies with balanced allele-specific copy number), allele-specific copy gain, and high-level focal amplification). While the mean overall megabase (MB) of SCA per NSAID user was lower than non-users (mean = 107.8 MB and 332 MB, respectively), a direct median test of total MB autosomal SCA between NSAID users and non-users was not significant (*p* = 0.10, median = 43.2 MB and 58 MB, respectively). With analysis by chromosome arm, NSAID users had significantly lower overall SCA load (*p* = 0.027), with varying levels of significance when analyzed by each SCA type (focal amplification, *p* = 0.01; cnLOH, *p* = 0.03; copy gain, *p* = 0.06; balanced gain, *p* = 0.065; copy loss, *p* = 0.78; HD, *p* = 0.84; see “Methods,” Additional file 2: Tables S15–S17, Additional file 3: Figures S4 and S5). Using a similar analysis for the number of SCA segments per chromosome arm with normalization of the data by arm length, NSAID users had significantly fewer overall SCA segments compared to non-users (*p* = 0.021) and fewer SCA segments in some individual SCA types (focal amplifications, *p* = 0.007; copy gains, *p* = 0.045; balanced gains, *p* = 0.05; HD, p = 0.05; copy loss, *p* = 0.17; cnLOH, *p* = 0.51). Taken together, these results suggest that NSAID use provides selective pressure primarily against copy gain events such as amplification and WGD.

Focal amplifications were not detected in any NSAID users (0/41) but were detected at a significantly higher frequency in NSAID non-users (8/41) (*p* = 0.03, Fisher’s exact, Additional file 2: Tables S18 and S19). Focal amplifications spanned oncogenes including tyrosine receptor kinases *ERBB2* and *MET*, and cell cycle regulators *CCND1*, *CCNE1*, and *CDK6*, which have all been reported as frequently amplified genes in EA [5, 11–15, 35]. The mean number of focal amplification segments was 0.54 per non-user, which is within the range of recently reported average levels of focal amplification in non-dysplastic and dysplastic BE adjacent to EA (0.42 and 0.91 segments, respectively) [14]. Genome doubling was not detected in any NSAID users, whereas four non-users had evidence of genome doubling. Taken together, the SCA results extend previous findings [21, 24] that NSAID use promotes an environment in the neoplasm that selects against development of or expansion of cells with cancer-promoting chromosomal alterations such as high-level focal amplification and genome doubling.

## Discussion

Use of aspirin and other NSAIDs has been associated with a reduction in cancer incidence and mortality for a number of cancer types, including EA. We hypothesized that NSAIDs exert their protective effects in part by reducing evolution of somatic mutations and lowering mutation diversity during neoplastic evolution in BE. In this study, we found that NSAID use decreased the somatic mutation load across the spectrum of mutations, interacted with smoking to alter mutagenic signatures, selectively reduced functional gene mutations, and lowered diversity of mutated genes in pathways critical for maintaining genomic integrity. These data extend previous findings showing the cancer risk reduction effect of NSAID use at the population level [7–9, 53] and in longitudinal studies of BE [20, 21, 24] to further characterize the impact of NSAIDs as protective agents at the DNA sequence level. Our results show that NSAID use reduced overall incidence of mutations and further supports a model in which cells with potential cancer-promoting mutations survive better in the absence of NSAIDs, leading to increased opportunity for selection and generation of genetic diversity in BE. The results enhance our understanding of how exposure to protective (e.g. aspirin, NSAIDs) and mutagenic (e.g. tobacco) factors interact to modulate the mutational landscape on which selection can act during neoplastic evolution and provides insight into the evolutionary mechanism by which aspirin and other NSAIDs can increase prevention of EA.

Some cancer types are characterized by mutational signatures that are dominated by exposure to particular mutagens—e.g. aflatoxin B_1_ or hepatitis B virus in hepatocellular carcinoma [54], tobacco exposure in lung cancer [55], and ultraviolet light in cancers of the skin [56]—while other cancer types appear to have complex mutational signatures that reflect multiple mutagenic processes occurring over the lifetime of an individual [56, 57]. Analysis of mutations in the context of their 5′ and 3′ neighboring bases can reveal the etiology of mutagenic processes, representing an integration of endogenous and exogenous mutational exposures with DNA damage recognition and repair processes [33]. In addition to mutation signatures S1 and S17, previous studies have identified signatures S2 (APOBEC), S3 (BRCA 1/2), and S18 (gastric/neuroblastoma) associated with EA (reviewed in [17]); however, in our study, these signatures only contributed a small fraction of mutations per patient in a subset of patients (Additional file 2: Table S10). In contrast, S1 and S17 comprise > 50% of all point mutations, confirming previous sequencing studies of BE and EA [5, 10–14, 16] and further advancing our understanding of how NSAID use prevents EA by showing that a combination of NSAID use and never smoking reduces the mutation signature S17 that is common in EA.

This WES study was designed to investigate somatic mutations from participants with detailed NSAID and cigarette use information, allowing for refined characterization of the somatic mutational landscape with consideration of a protective intervention in combination with a mutagenic exposure. Tobacco use is known to increase point mutations [30] and is an established risk factor for EA [58, 59]. BE tissue is exposed to a variety of mutagens including those that may be swallowed in cigarette smokers [6] and in those found in the intrinsic injurious mutagenic environment of bile and acid reflux in the esophagus that is common in individuals with BE [3, 5, 18]. Our results show that NSAID use reduced overall mutations, while smoking specifically increased S17 mutations in BE. Mutational signatures attributed to tobacco use vary across cancer types. Consistent with what has been shown in EAs in smokers [30], we did not detect the lung smoking signature (S4) in smokers in this study. While Alexandrov et al. [30] did not detect a significant difference in any mutation signatures between smokers and non-smokers in EA, our current study measured the mutation spectrum at an earlier stage in BE, before widespread chromosomal alterations found in EA, potentially allowing for discrimination of the contributions of NSAIDs to repress mutant cells in combination with the signature-specific increase in S17 mutations with tobacco use.

Modulation by NSAIDs of DNA adduct formation from passive tobacco smoke has been observed in mice [60] and an interaction between aspirin and smoking has also been observed in the colon, where reduction of incidence of polyps by daily aspirin use has been shown to be abrogated in active smokers [61]. While smoking rates in the general United States population have decreased over the past 35 years [62], the incidence of EA has increased [63, 64], suggesting that while tobacco exposure is a risk factor for EA [59, 65], the effect of smoking is only one factor in the complex environment to which the BE tissue is exposed. S17 was the predominant mutation signature in this study (31.5% of all mutations by deconstructSigs), but the majority of mutations were not classified in S17 and additional mutation types such as indels were not included in this signature analysis.

Our finding that NSAID users had significantly fewer mutations with a high variant allele frequency (VAF > 0.3) is consistent with NSAID use reducing expansion of cell populations with potential cancer promoting mutations. The samples sequenced in this study comprised > 98% purified BE epithelium [27], making VAF in diploid regions a close approximation of clonal prevalence in the bulk epithelium. High VAF can indicate genetic alterations with a strong selective advantage in a given tissue [66]. NSAID use may slow expansion or increase elimination of mutant cells or some combination of the two. A recent study in colorectal carcinoma cell lines [67] reported aspirin use at physiological levels slightly reduced overall cell growth rate and increased cell death rate, potentially reducing the expansion of clonal populations with genomic alterations, consistent with our findings. Future studies using multi-region sequencing are required to determine if NSAID use lowers the diversity of point mutations throughout the BE segment within an individual patient [68].

The spectrum of mutated genes found in NSAID users and non-users was highly heterogeneous, consistent with previous WGS and WES studies in EA that found very few genes mutated at high frequencies across cancers, with the exception of *TP53* [5, 10–14, 16]. Given the rarity of individual genes mutated at high frequency, pathway analyses have been used to elucidate perturbations in critical cellular processes in cancer [13, 69–71]. Using this approach, nine of 35 pathways had significantly fewer functional mutations in NSAID users compared to non-users, with an average of 50% reduction in mutations in each pathway. Functional mutations in these nine pathways were significantly lower in NSAID users, while non-functional mutations were not significantly different between users and non-users except in the *TP53* pathway. This suggests that NSAID use provides selection against cells with functional mutations in these pathways or suppresses expansion of mutant clones and supports a model where, in addition to a general reduction of mutations, NSAID use selects against development or expansion of cell populations in which maintenance of a stable genome has been compromised by somatic mutations in genes important in the evolution to cancer.

Diversity measures of somatic chromosomal alterations in BE have been repeatedly shown to be robust predictors of progression to EA [27, 72–74]. Given the potential genetic heterogeneity across the Barrett’s epithelium in any one patient [27, 68, 72, 73], it is possible that other samples in the esophagus may have a different mutation spectrum. In this study, for each participant, we examined one biopsy in the middle of the Barrett’s segment and multiple samples over time are needed to accurately assess the effect of NSAID use on mutation rate and diversity throughout the BE segment. Despite these limitations, our findings that NSAID use significantly lowers diversity of mutations across key cancer-associated pathways suggest the mechanism by which NSAID use protects against cancer incidence and mortality is in part through lowering diversity of mutations that fuels evolutionary selection to drive cells toward cancer [1].

It is well-established that most EAs develop through the inactivation of *TP53* and subsequent genomic instability (typically involving tetraploidy, aneuploidy and often via complex mechanisms such as chromothripsis [5, 17, 75]), but to our knowledge, there is little known about the direct effect of inactivation of *TP53* on the rate of point mutations genome-wide. In our study, individuals with *TP53* gene mutations had an increased mutational load compared to those with WT *TP53*, but NSAID use decreased mutations across the 96 trinucleotide substitutions regardless of *TP53* status. Recent studies show that *TP53* mutant cancers have an increase in mutation rate beyond that expected from simply aging alone [76], suggesting either that a high mutation rate increases the chance of *TP53* mutation, or that mutation of *TP53* promotes genomic instability that in turn increases mutation rate. Our results show that even when including *TP53* mutation and smoking status in the statistical model, NSAID users had lower overall mutation load than non-users. It has been shown that aspirin can eliminate tetraploid cells in cell culture and reduce the accumulation of tetraploid cells in APC(Min/+) mice [77]. In addition, cells that develop complex karyotypes due to abnormal chromosomal segregation have been shown to undergo removal through immune surveillance [78]. Inhibition of COX-2 by celecoxib also has been shown to enhance the efficacy of anti-PD1 immunotherapy through suppression of PGE_2_ [79]. Thus, NSAID use either creates an environment in which fewer somatic genomic alterations are being generated and/or an environment in which cell populations with increased genetic alterations are selected against. Since a cancer endpoint was not evaluated in this study, it is not possible to determine if NSAID users who do progress to cancer develop cancers with different mutational burden/profile than cancers arising in non-users. The results of this study suggest that applying molecular pathological epidemiological (MPE) approaches to future studies of NSAIDs and cancer may be used to refine the effect size by sub-classifying BE patients by molecular pathologic features to provide stronger evidence of causality than analysis of overall disease [80–82].

Significantly less SCA was found in NSAID users, with focal high-level copy number amplifications and genome doublings only found in NSAID non-users. This is consistent with a model where NSAID use results in a selection against cell populations having increased levels of genomic alterations, especially gain events. An earlier study by Kostadinov et al. found that NSAID use significantly reduced the rate of acquisition or rate of accumulation of SCA by tenfold [24] and suggested that “NSAIDs may prevent the occurrence of massive numbers of [SCA] on single lineages (branches of the phylogeny) or limit the clonal expansion of such lineages.” Only four of the 82 participants in this study were also evaluated by Kostadinov, thus validating the results of the earlier study in an independent patient set using orthogonal technology (WES vs SNP arrays).

The COX-1/2 pathway is classically implicated in the cellular response to NSAIDs and the direct inhibition of the COX-2 enzyme by NSAIDs has been extensively studied [9]. While our results show lower mutation load in NSAID users, the mechanisms by which this would occur via these established pathways is unclear. Reduction of the inflammatory response, particularly prostaglandin E_2_ (PGE_2_) and its subsequent roles in potentiating Wnt signaling and angiogenesis [83, 84], along with reduction of oxidative damage likely play a role in the anti-cancer properties of NSAID use. Under normal physiological conditions in many cell types (e.g. cardiac and endothelial cells), NSAIDs can induce reactive oxygen species production and increase apoptosis rates, leading to cell death [85]. However, under conditions of chronic inflammation, NSAIDs reduce overall inflammation through inhibition of PGE_2_ synthesis and PGE_2_ promotes apoptosis resistance, angiogenesis, and neoplastic progression [86]. Germline genetic variants have been associated with BE, particularly the Cox pathway gene *MGST1*, which has been suggested to function to counteract insults from reflux and cigarette toxins [87]. While we found no difference in mutation load between NSAID users and non-users (evaluating functional or non-functional mutations) across genes that regulate COX-2 expression, larger studies that integrate inherited variants in inflammatory pathway genes and somatic mutations are warranted.

Methods developed in the emerging field of MPE can “…enhance casual inference by linking putative etiological factors to specific molecular biomarkers as outcomes” [82]. Our study has characteristics of an MPE study in that: (1) our patients were drawn from a larger prospective cohort study; (2) we are testing the effect of a potential etiologic factor on specific molecular characteristics of the disease of interest; and (3) the study was designed to demonstrate a relationship between an exposure and specific molecular alterations and identify disease subtypes that associate with benefits from lifestyle or pharmacological intervention [80, 82]. There are limitations to this study. This study was specifically designed to evaluate somatic mutations in neoplastic tissue after periods of NSAID use or non-use and was not designed to study the effect of NSAID use on progression to EA. Thus, progression to a cancer endpoint is not considered in the study design nor included in any analyses. Due to cost constraints of generating 80× exome sequences, this study was also limited in the number of samples per patient sequenced and the number of individuals in the study, when compared to the number of participants in population-based analyses examining the role of aspirin or NSAID use on cancer incidence and mortality. However, our longitudinal cohort of individuals with documented NSAID use over time allowed us to examine the effects of NSAID use on the genome and was sufficiently powered to detect differences in somatic mutation levels between NSAID users and non-users. WES does not allow analysis of structural alterations or mutations in non-coding regions outside the genomic regions captured by the library preparation method. However, the capture method used in this study included 32 Mb of upstream and downstream sequence surrounding coding regions, which allowed comparison of somatic SNVs and indels in both coding and non-coding regions of the genome. Participants were required to have at least two endoscopies; therefore, those who left the cohort (e.g. intervention, death) after the initial endoscopy were not included and may have different results from those who remained in the cohort. Validation of these results would ideally be performed in an independent study examining the role of NSAID use in reducing risk of progression to EA, such as the ongoing AspECT trial [88].

## Conclusions

In conclusion, this study supports a model in which mutant cells survive better in the absence of NSAIDs. NSAID use either reduces the frequency and diversity of somatic mutations and chromosomal alterations and/or increases the likelihood that mutated cells will be removed from cell populations. Our finding that NSAID users who never smoked have lower S17 mutations provides insight into the complex factors contributing to the mutational spectrum in BE. This study additionally suggests that functional somatic gene mutations in key cancer-associated pathways are selected against when using NSAIDs. Our findings provide insight into cancer prevention efforts and informs future studies to integrate precision prevention approaches to EA interception and early cancer detection [89–91].

## Additional files

Additional file 1: Supplemental Methods: Additional file 1 describes details of the study subjects, study design, exposure quantification methods, sample processing and sequencing methodologies, statistical methods for all analyses, and copy number/cnLOH calling methods. (DOCX 88 kb)

Additional file 2: Supplemental Tables: Additional file 2 contains Supplemental **Tables S1-S21.** (XLSX 4872 kb)

Additional file 3: Supplemental Figures: Additional file 3 includes Supplemental **Figures S1-S7.** (PDF 1067 kb)

## Abbreviations

BE: Barrett’s esophagus
cnLOH: Copy neutral loss of heterozygosity
EA: Esophageal adenocarcinoma
HD: Homozygous deletion
NMF: Non-negative matrix factorization
NSAIDs: Non-steriodal anti-inflammatory drugs
SCA: Somatic chromosomal alterations
SNV: Single nucleotide variant
VAF: Variant allele frequency
WES: Whole exome sequencing
WGD: Whole genome doubling
WGS: Whole genome sequencing
