# Correction to: NSAID use and somatic exomic mutations in Barrett’s esophagus

**DOI:** 10.1186/s13073-019-0625-y

**Published:** 2019-03-12

**Authors:** Patricia C. Galipeau, Kenji M. Oman, Thomas G. Paulson, Carissa A. Sanchez, Qing Zhang, Jerry A. Marty, Jeffrey J. Delrow, Mary K. Kuhner, Thomas L. Vaughan, Brian J. Reid, Xiaohong Li

**Affiliations:** 1 Division of Human Biology, Fred Hutchinson Cancer Research Center, PO Box 19024, 1100 Fairview Ave N, Seattle, WA 98109-1024 USA; 2 Bioinformatics Shared Resource, Fred Hutchinson Cancer Research Center, PO Box 19024, Seattle, WA 98109-1024 USA; 3 Genomics Shared Resource, Fred Hutchinson Cancer Research Center, PO Box 19024, Seattle, WA 98109-1024 USA; 4 Genomics and Bioinformatics Shared Resources, Fred Hutchinson Cancer Research Center, PO Box 19024, Seattle, WA 98109-1024 USA; 5 Department of Genome Sciences, University of Washington, Foege Building S-250, Box 355065, 3720 15th Ave NE, Seattle, WA 98195-5065 USA; 6 Department of Epidemiology, University of Washington, Division of Public Health Sciences, Fred Hutchinson Cancer Research Center, PO Box 19024, Seattle, WA 98109-1024 USA; 7 Department of Medicine, University of Washington, Division of Human Biology, Fred Hutchinson Cancer Research Center, PO Box 19024, Seattle, WA 98109-1024 USA

**Correction to: Genome Med**

**https://doi.org/10.1186/s13073-018-0520-y**

It was highlighted that in the original article [1] the Availability of data and materials section was incorrect. This Correction article shows the revised Availability of data and materials section.

**Availability of data and materials**

The datasets generated during the current study are available in the database of Genotypes and Phenotypes (dbGaP) repository, study accession ID phs001654.v1.p1

## References

[1] Galipeau (2018). NSAID use and somatic exomic mutations in Barrett’s esophagus. Genome Med.

