# Supplementary material for: NSAID use and somatic exomic mutations in Barrett’s esophagus
Source: Genome Med. 2018 Feb 27;10:17. doi: 10.1186/s13073-018-0520-y (PMC5830331; doi:10.1186/s13073-018-0520-y)
Supplement: Supplementary file 1 — Supplemental Methods: Additional file 1 describes details of the study subjects, study design, exposure quantification methods, sample processing and sequencing methodologies, statistical methods for all analyses, and copy number/cnLOH calling methods. (DOCX 88 kb) [file 13073_2018_520_MOESM1_ESM.docx]

**Additional File 1: Supplemental Methods**

**Study subjects**

The Seattle Barrett’s Esophagus Study has been approved by the Fred Hutchinson Cancer Research Center Institutional Review Board IRB #8344. Participants enrolled in the study signed research consent. Participants with endoscopic and histologic BE without EA met the inclusion criteria for the research study and underwent endoscopic biopsy surveillance for early detection of EA [1]. Blood samples were obtained and processed for use as constitutive genome controls. Participants provided detailed medical history and history of specific prescription and over-the-counter medications [2].

This study cohort included 408 individuals (Additional file 3: Figure S1). All biopsies in the study were collected between 13 February 1995 and 25 October 2011. 129 eligible participants included those with at least two endoscopies (prior to EA diagnosis) and with consistent NSAID use or nonuse. 34 were excluded due to only one endoscopy before EA diagnosis (N=4) or due to an external event including endoscopic mucosal resection and/or ablation during follow-up (N=30). The remaining 374 participants were classified as consistent NSAID users (N=87), inconsistent NSAID users (N=221), or consistent NSAID nonusers (N=66). Of the consistent NSAID users and nonusers, 24 participants were excluded due to focal or minimal Barrett’s epithelium. This resulted in 72 consistent NSAID users and 57 consistent NSAID nonusers.

**Study design**

From 129 eligible participants, a cross-sectional study was designed from all 72 NSAID users and 57 nonusers. Each NSAID user was randomly selected to a matched NSAID nonuser based on sex, age at baseline (within 10 years) and smoking status (never/ever across all endoscopies). 41 NSAID user:nonuser pairs met this matching criteria (Additional file 2: Table S2). Using metaplastic (Barrett’s epithelium) samples only, this study design is similar to the “case-case” approach described in molecular pathological epidemiology with subtyping being defined by the exposure (NSAID use) and somatic mutations as the molecular feature outcome [3]. Of these 41 pairs, NSAID users had 2 or more (mean 5, range 2-17) consecutive endoscopies of NSAID use spanning an average of 5.6 years (range 0.6-15.5). NSAID nonusers had 2 or more (mean=4, range 2-10) consecutive endoscopies of no NSAID use spanning an average of 3.6 years (range 0.5-10.6).

For the endoscopy at time of WES, all patients had a histological diagnosis of intestinal metaplasia (N=45), indefinite for dysplasia (N=10), low-grade dysplasia (N=9) or high-grade dysplasia (N=18) without histologic evidence of cancer. For each patient, somatic genomes from one BE sample taken from the middle of the BE segment and their matched constitutive blood control were analyzed by WES. Endoscopic biopsies were selected from the middle-most level within histologically classified BE between the visualized squamocolumnar junction and the gastroesophageal junction.

### **Aspirin and NSAID use and smoking status**

All participants were interviewed in the clinic on the same day as endoscopy [4]. Information on past and present medication and smoking use was obtained [5]. Use of prescription and over-the-counter drugs containing aspirin or other NSAID data was collected in a questionnaire modeled on the U.S. collaborative case-control study of EA, as previously described [6, 7].

Participants were defined as NSAID users if they consistently reported at each interview during the assessed time-period that they had been using aspirin or other NSAIDs at least once a week for six months or more (Additional file 2: Table S2 for NSAID use/nonuse duration). Of the 41 NSAID users, 20 reported using aspirin only, whereas 21 reported aspirin and/or other NSAIDs. None of the NSAID users were taking aspirin or other NSAIDs for cancer prevention. Of the 41 NSAID users, at their baseline, 10 were using prescribed non-aspirin NSAID medication for pain relief, and the remaining 31 were taking over-the-counter aspirin for prevention of cardiovascular disease (N=16), pain relief (N=5), for both indications (N=7) or other reasons (N=3). 32 NSAID medications were included in the questionnaire, therefore the dose of each medication was not evaluated. Participants were defined as NSAID nonusers if they consistently reported at each interview during the assessed time-period that they had *not* been using aspirin or other NSAIDs at least once a week for six months or more. Participants who reported variable aspirin/NSAID use across interviews or former use during the assessed time-period were excluded (Additional file 3: Figure S1). Participants were defined as ever smokers if they had reported smoking at least one cigarette/day for six months or more at any interview/endoscopy, and were defined as never smokers only if they had reported never smoking at least one cigarette/day for six months or more at any interview.

**Sample processing**

Epithelial isolation of endoscopic biopsies was performed as previously described [8]. DNA was extracted using PureLink Genomic DNA Mini Kit (Invitrogen/Life Technologies) according to manufacturer’s protocols and quantitated with Picogreen (Quant-IT dsDNA Assay, Invitrogen/Life Technologies). DNA from a constitutive blood sample for each patient was run in the same flow cell lane as the matched DNA from BE tissue for that patient.

**Pre-capture KAPA library preparation**

164 pre-capture sequencing libraries were each prepared from 1µg of intact genomic DNA. Genomic DNA was fragmented on a Covaris LE220 Focused-ultrasonicator (Covaris, Woburn, MA, USA) using factory settings for an average size of 300 bp and prepared using the KAPA DNA Library Preparation and HiFi PCR Kits (Kapa Biosystems, Wilmington, MA, USA) on a PerkinElmer Sciclone NGSx Workstation (PerkinElmer, Waltham, MA, USA). Libraries size distributions were validated using an Agilent 2200 TapeStation (Agilent Technologies, Santa Clara, CA, USA) and quantified with a Caliper/PerkinElmer LabChip DS spectrophotometer (PerkinElmer, Waltham, MA, USA).

**Exome capture and sequencing**

Individually indexed KAPA libraries were hybridized to the NimbleGen SeqCap EZ Exome + UTR capture probe set (Roche NimbleGen, Madison, WI, USA) according to the manufacturer’s protocol utilizing a one capture per library strategy. This library captures 64 Mb of coding exons and miRNA regions plus 32 Mb of expanded coverage of 5’- and 3’-untranslated regions (UTRs). Post hybridization NimbleGen bead washes and captures were performed on a Sciclone NGSx Workstation, post capture libraries were amplified using 14 cycles of PCR using the KAPA HiFi PCR kit. Libraries size distributions were validated using an Agilent 2200 TapeStation and quantified with a Caliper/PerkinElmer LabChip DS spectrophotometer. Additional QC, blending of pooled indexed libraries, and cluster optimization was performed using Life Technologies’ Invitrogen Qubit® 2.0 Fluorometer (Life Technologies-Invitrogen, Carlsbad, CA, USA). Individual indexed libraries were pooled (24-plex) and clustered onto Illumina v4 flow cells (Illumina, Inc., San Diego, CA USA) using an Illumina cBot. Sequencing was performed using an Illumina HiSeq 2500 in high-output mode using v4 reagents, and employed a paired-end, 100 base read length (PE100) sequencing strategy. Each BE and corresponding normal paired sample were verified as deriving from the same patient based on genotyping data as described previously [8], (Additional file 3: Figure S6).

**Sequence alignment, metrics, and somatic mutation calls**

Paired-end short reads were first aligned to the human genome reference assembly (GRCh37/hg19) using Burrows-Wheeler Aligner (BWA, v0.7.10) [9]. Average depth of coverage was 82X (range 59.76-157.75 depth of coverage) (Additional file 2: Table S20, Additional file 3: Figure S7). The overview of sequence alignment statistics was computed for each sample using flagstat of Samtools [10].The resulting alignment data were further processed with the best practice of Genome Analysis Toolkit (GATK, v3.3) [11, 12]. Sequence coverage was computed for each sample using DepthOfCoverage of GATK. Somatic point mutations were detected on paired normal and BE samples using muTect [13], and Strelka [14]. The intersection of point mutations identified by each caller was used in subsequent analyses (28,430 total mutations). 1,655 somatic indels were identified in paired BE samples using Strelka.

**Frequency analysis of point mutation sites in their tri-nucleotide context and overall mutation load**

The 5’ and 3’ flanking nucleotides of each SNV were determined by read coverage in the paired normal (blood) BAM files. Reads overlapping each SNV position were examined, and flanking 5’ and 3’ nucleotides with phred quality score >=20 were counted per SNV site. The modal nucleotide base at each flanking position was used as a part of the tri-nucleotide sequence. Mutations in their tri-nucleotide context were then classified according to the six substitution subtypes referred to by the pyrimidine of the mutated Watson-Crick base pair, resulting in 96 mutation types. The number of mutations in each of these tri-nucleotide contexts was counted separately for each patient and was normalized by the frequency of these tri-nucleotides in the exome capture region (with 200 base extensions surrounding each of the exomes) in hg19. The overall difference in tri-nucleotide frequencies was compared between NSAID users and nonusers using a sign test on the differences in median values across each of the 96 tri-nucleotides. Additionally, a direct comparison of total mutation counts (SNVs and indels) between NSAID users and nonusers was made using the Kruskal-Wallis test to avoid possible outlier effects [15].

**Mutation annotation for gene functional impact**

Each SNV and indel was assigned a functional impact on RefSeq transcripts [16] (annotations downloaded 14 December 2015 from the UCSC Table Browser [17]) and binned into 15 mutation classes. These classes included exon-synonymous, exon-nonsynonymous, intron, 5’UTR-exon, 5’UTR-intron, 3’UTR-exon, 3’UTR-intron, ncRNA-exon, ncRNA-intron, downstream, upstream, coding-splicing, UTR-splicing, ncRNA-splicing, and intergenic. Mutations were defined as “functional” or presumed likely deleterious in the following categories: non-synonymous coding mutations, those within 5’ or 3’UTR exons, and those affecting splicing (within 2 bps of a splice junction). All other mutations within 1 kb of a transcript were also associated with the transcript as “non-functional” mutations. Due to cell transcript usage ambiguity, only genes where all transcripts had at least one “functional” mutation were used for further analysis of genes deleteriously impacted by mutation, and those considered not functionally impacted were those not having any functional mutations in any of their transcripts.

**Additional statistical tests for overall mutation load**

A trend test of the mutation load (combined count of SNVs and indels per patient) between NSAID users and nonusers was implemented with the following three steps: first, the median mutation loads for users and nonusers for each functional group were determined. Second, the differences between users and nonusers for each of these medians were calculated, resulting in a distribution of differences with values from each of the different functional groups. Finally, a sign test was used to test if this distribution has zero median differences, compared against the alternative hypothesis that the distribution does not have zero median differences [18].

As the somatic mutation (SNV/Indel) distribution in the genome may be heterogeneous within a given sample (e.g., some chromosome arms might have large numbers of mutations while others have low mutation load), quantifying the mutation load by chromosome arm was more efficient than using only one quantity for mutation load analysis such as comparing the median or mean mutation load per sample. Thus to compare mutation density (functional only or non-functional only), the mutation load was calculated per chromosome arm and normalized by arm length. A mixed effects model was then used to test if NSAIDs affect the mutation load [19]. Specifically, mutation density of each arm in a genome was treated as repeated measurements for a given patient, with NSAID use considered as a fixed effect. Individual subjects were treated as sources of the random effects in the regression model.

***TP53* mutation and NSAID effect on mutation load**

Mutation load (total SNVs and indels per patient) was compared in NSAID users vs nonusers using a Kruskal-Wallis test for those individuals with a *TP53* mutation. Similar to before (see section “Frequency analysis of point mutation sites in their tri-nucleotide context and overall mutation load”), a sign test was performed on differences in medians across the 96 tri-nucleotide context of SNVs in NSAID users and nonusers for individuals both with and without a *TP53* mutation. Finally, a multivariable regression model was used to test the effect of NSAID use on total mutations. Specifically, *TP53* mutation status, NSAID use status, and smoking history (all binary variables) and their interaction terms were used as independent variables with total mutation count as a dependent variable; a multivariable regression model was used to evaluate the significance of *TP53* mutation status, NSAID use, and smoking. Total functional mutation load was used as a dependent variable in a similar analysis.

**Variant allele frequency comparison**

VAF is a continuous value measuring the proportion of a variant, such as a point mutation, in total reads at a given variant site. To test if NSAID use suppresses mutated cell populations, somatic SNV VAFs were compared between NSAID users and nonusers for both total SNVs, as well as functional SNVs. Since chromosome copy number can affect the VAF values, this comparison was carried out only in diploid chromosomal regions without any SCA (see “Somatic chromosome copy number and LOH estimation” section). Specifically, all SNVs were first divided into seven bins based on VAF (VAF < 0.1, 0.1-0.2, 0.2-0.3, 0.3-0.4, 0.4-0.5, 0.5-0.6, >=0.6) and the numbers of mutations were counted in each bin for each patient. Then, patient NSAID usage and the VAF bin categories were treated as main effects in ANOVA analysis to test if NSAID usage significantly reduced the VAF values. To test the significant portion of the VAF bins, starting from all seven bins, lower VAF bins were iteratively dropped and the remaining values re-tested to determine the VAF threshold where an NSAID effect was observed (where the NSAID effect reached statistical significance and maintained significance with further bins dropped). The remaining bins identified VAF values significantly affected by NSAIDs. Additionally, this same method was performed on normalized mutation counts, where the number of SNVs in each bin were first divided by the total number of SNVs for a patient, with subsequent steps being identical.

**Mutation signature discovery**

Mutational signatures were explored using an implementation of the approach developed by Alexandrov, et al., using non-negative matrix factorization [20] to *de novo* decipher mutational signatures across all participants in the study. Nones et al. found five mutation signatures in WGS [21], therefore, our analysis constrained the initial signature search to five, and then iteratively decreased the number of allowable signatures until remaining signatures were of high confidence with scores ≥ 0.9. This resulted in two high-confidence signatures consistent with COSMIC Signatures 1 and 17 [22].

Although the method above is a powerful *de novo* signature discovery tool, it is most effective with large samples sizes. As such, deconstructSigs [23], which utilizes a multiple linear regression model to fit known signatures [22], was used to evaluate each patient’s mutational profile, thereby identifying contributions of the different signatures in individual patients. deconstructSigs was run using default parameters with sample mutational profiles normalized to WGS tri-nucleotide context values to make them comparable with the COSMIC signatures.

**Gene-by-gene mutation analysis**

Total functional mutations (see definition in Section “Mutation annotation for gene functional impact”) per individual were determined, and NSAID users were compared to nonusers using a Kruskal-Wallis test. Genes were first selected with a threshold of p≤0.2 [24]. Then genes significantly affected by NSAID use were determined with a relatively stringent threshold of having a minimum of 5 individuals with a functional mutation in the gene (users and nonusers combined), and having a FDR (false discovery rate) = 0.1. A less stringent threshold of selecting genes with FDR = 0.2 was also used. The FDR multiple testing adjustment was performed on these genes using the Benjamini-Hochberg procedure [25]. Briefly, for a given FDR threshold *q*, all the *m* p-values in a comparison set were ranked from small to largest. We found the largest rank *i* such that $m*p_{\left( i \right)}/i\leq q$, and all p-values with lower or equal rank were taken to be significant.

**Pathway selection/ processing and gene name verification**

Pathway information was downloaded from the Network Data Exchange’s database of pathways [26]; 25 from the NCI-Pathway Interaction Database [27], 11 from the Reactome Pathway Database [28, 29] (see Additional file 2: Tables S1). Pathways were selected to represent the main cellular processes in housekeeping, as well as those of interest in cancer development. Of the 4,172 gene names directly taken from these pathways, only 71 did not match HUGO [30] gene names from the RefSeq list mentioned earlier (see Section “Mutation annotation for gene functional impact” in Methods). Some of these were determined to be newer versions of the RefSeq names. Therefore, using Genenames.org [31] to generate a list of RefSeq gene name aliases/conversions (using the fields: “Approved Symbol”, “RefSeq IDs”, and “RefSeq (supplied by NCBI)”, ignoring names with “Withdrawn” symbols, downloaded October 27, 2016), 36 gene names were converted to the old RefSeq counterparts (consistent with the rest of our analysis from other sections), leaving only 35 gene names that could not be converted. These were dropped from further analysis, and the 4,137 gene names that either appeared directly to match RefSeq, or were converted to our RefSeq names were utilized. Finally, the “DNA Replication” and “Mitotic M-MG1 phases” pathways were found to share all the same gene names except for one each, so a combined pathway “DNA_Replication.Mitotic_M-MG1_phases” was created for analysis using the union of genes from each of these two pathways, with the two separate pathways dropped from further analysis. Additionally, one pathway was curated from Kang et at., [32] (Additional File 2: Table S21).

**Pathway mutation diversity assessment**

Pathway mutation diversity quantifies the number of pathways affected by point mutations and indels, e.g., for a given total number of mutations, the more pathways these mutations affect, the higher the pathway mutation diversity compared to the same number of total mutations affecting fewer pathways. The Shannon index was modified to assess this diversity, utilizing an additional normalization to account for the different number of genes involved in each pathway. We assumed that for a given number of mutations occurring in a pathway, their overall impact will be larger for a pathway with fewer genes: e.g., 10 mutations occurring in a pathway of 20 genes will have a greater biological impact than 10 mutations in a pathway of 1000. Thus, we let $n_{i}$ be the number of genes annotated in the $i$*-*th pathway, with $q$ total pathways. Let $m_{i}$ be the observed number of mutations in $i$-th pathway, and ${m'}_{i}$ be the normalized number of mutations for $i$-th pathway. Therefore, ${m'}_{i}=m_{i}/ n_{i}$. The modified Shannon index $H'= -\sum_{i=1}^{q} p_{i}\ln p_{i}$ was then calculated with $p_{i} = {m'}_{i} / \sum_{i=1}^{q} {m'}_{i}$. To account for the number of pathways investigated, $H'$ was normalized as $H'/\ln q$. A Kruskal-Wallis test was used to compare the difference of the diversity of pathway mutations quantified by the modified Shannon index between users and nonusers.

**Somatic chromosome copy number and LOH estimation**

***ADTEx vs 1M Illumina SNP array***

12 DNA samples in this study had been run on 1M Illumina SNP arrays in a previous study [8]. SNP arrays are considered to be a robust platform for SCA measurements [33], providing for this study a point of comparison as a robust standard. For this study, ADTEx v2.0 [34] was run both with and without the “estimatePloidy” option to obtain SCA calls, but many SCA events were missed upon comparison with the SNP array, necessitating custom processing for more accurate SCA calls.

***Apply ADTEx/ generate minimum sized segments***

BAFs were extracted from MuTect results for all positions assessed (not just mutations passed). ADTEx v2.0 was modified to fix a bug (see <https://sourceforge.net/p/adtex/discussion/general/thread/eeaad82b/>), then run twice using the extracted BAFs and BAM files for all 82 paired patient samples: once with all default parameters, and once with the additional option of "--estimatePloidy". Segmentation from ADTEx was defined from two of its output files. DOC segmentation (from cnv.result files) were determined from the "CNV_start" and "CNV_end" fields, while BAF segmentation (from zygosity.res files) were determined from consecutively identical calls made to the "zygosity" field. Segmentation boundaries were combined from both the DOC and BAF segments, from both runs of ADTEx, leading to boundaries being merged from four sources, leading to minimally sized genomic segments for each sample. Also, any segments spanning a centromere were broken into two, removing the centromeric region as defined by the exon capture regions. For each of these minimally sized segments, DOC data points (from the "rationormalized_after_smoothing" field for each exon considered in cnv.result files) and BAF data points (from the "tumor_BAF" field of the zygosity.res files) were assigned, and utilized in further processing.

***Combining neighboring segments***

Segments were then combined with their neighbors based on the following criteria. 1) Consecutive segments ≥ 1 Mb apart were excluded from being considered for merging. 2) Segments with less than 30 DOC data points were automatically merged with the neighbor with the closest average DOC value. 3) If the difference in the DOC means between consecutive segments was less than one standard deviation (from either segments' DOC distribution), then the two segments may be merged -- however, if neighbors on both sides meet this criteria, then the neighbor with the closer average DOC were merged with this segment. For 3), if there were >= 12 BAF data points with BAF values above and below 0.5 (so a minimum of 24 BAF values) for each segment, then also use a discrete wavelet transform of the BAF values to inform if the BAF distribution was split (away from 0.5 – an imbalanced number of each allele), or not split (around 0.5 – balanced number of each allele). In order for consecutive segments to be merged (having fulfilled condition 3), both segments must match in the split/not-split call. If there were insufficient BAF values to make the split/not-split call, then merging was strictly decided or not based on the DOC condition. The split/not-split calls were made once for each of the minimally sized segments before any merging, and once more after segments were merged (prior to constructing SCA classes).

***Generate SCA classes/ make SCA calls***

Similarly to the method used to combine neighboring segments, SCA classes were generated, combining similar segments from anywhere in the genome (comparing average DOC and BAF split/not-split calls). Classes were combined using these rules until the smallest number of classes could be generated. Then, SCA classes were ranked by average DOC. The largest (most number of DOC points) segment class was then called "Baseline" if it had a BAF not-split call for the class, and was called "copy neutral LOH" (CNLOH) if the BAFs were split. Adjacent classes were identified as the opposite if they would have been merged with the largest SCA class, except that the BAF calls were of the other type, preventing merging. Any SCA class with average DOC < 0.5 was identified as "Homozygous Deletion", regardless of the BAF call, and any other SCA classes with DOC < the "Baseline" or "CNLOH" classes were identified as "Copy Loss". SCA classes with average DOC ≥ 10 "Baseline" standard deviations from the "Baseline" class were identified as "High-Level Focal Amplification", regardless of the BAF distribution, and any remaining SCA classes in between the "Baseline" or "CNLOH" classes and the "High-Level Focal Amplification" classes were identified as "Allele-Specific Copy Gain" if the BAF distributions were split, and "Balanced Gain" if the BAF distributions were not split. All genomic segments that contributed to the SCA classes were assigned the SCA call of their parent class.

***Somatic genome copy number baseline verification and adjustment***

The final SCA calls for WES samples that additionally had SNP arrays were found to be consistent with the SNP array calls. All 82 patient SCA calls were visually inspected for accuracy, and four samples (participants 391, 163, 660, and 798) with evidence of genome doubling (> 50% of somatic genome had copy number gains – balanced gain, allele-specific copy gain, and high-level focal amplification) required manual re-base lining. The SCA classes, and their corresponding segments that made up the classes, were re-assigned SCA calls that were more consistent with manual interpretation of the data, which is a process that considers DOC and BAF values simultaneously so that both DOC and BAF match across the genome. This interpretation yields copy loss and gain called as deviations from a diploid 2N genome: that is, a region of three copies was assessed as a gain, even in a genome doubled sample.

***Merge consecutively identical SCA calls***

When examining a sample and its neighbors, there are instances where two consecutive segments do not merge (e.g. the mean depth of coverages are too far apart from each other, based on the spread of coverage in either segment). However, upon creation of the SCA classes, these consecutive segments may have been placed in the same class (since the class DOC spread will be broader, since it has DOC points from a collection of segments across the genome). As such, these previously un-merged consecutive segments may have the same SCA call associated with them. To account for this, in post SCA calls, consecutive segments called identically ("Baseline", "CNLOH", etc) were merged into larger segments if the two segments were <= 3 MB apart from one another.

***Small homozygous deletions***

Although there are several HD regions which are commonly observed in BE and EA (e.g. within *FHIT*, *CDKN2A* and *WWOX* gene regions), the majority were missed by the custom modified ADTEx SCA call algorithm due to their very small size (typically 1 to 200 kb). To obtain small HD information in all exome samples, we first used the 12 samples run both on array and exome-seq to identify HD regions called by the array (based on both the DOC and the BAF pattern). DOC of the exome data was collected from these 12 samples for the same HD regions as well other non-HD regions to be used as a training dataset to train a multivariable logistic regression model for the prediction of HD in other exome samples with no SNP array data. Leave-one-out cross validation showed that the trained predictor for HD is of >80% sensitivity and >99.9% specificity. These HD calls were then merged with the previous SCA calls.

**Somatic chromosome alteration (SCA) analysis**

A Kruskal-Wallis test was performed to compare overall SCA load across NSAID users and nonusers directly, as a point of comparison. However, due to the stochastic dynamics of the somatic genome (specifically, of SCA) in time and space, the SCA distribution in the genome is heterogeneous for a given sample, e.g. some chromosome arms might have large amounts of SCA while others may have low or no SCA. This suggested that using a normalized chromosome arm level of quantification would be more efficient than using the median or mean to represent the genome of a sample for SCA assessment. A mixed effects model [35] was used to assess the effect of NSAID use on the SCA load from various types of SCA (total MB of SCA, as well as each SCA type from above, separately). Specifically, for a given type of SCA, SCA loading in each chromosome arm (normalized by arm length) were treated as repeated measurements for a given patient with a test performed on the effect of NSAID use on the various types of SCA load. An analogous analysis was also performed for the number of SCA segments of each type. Fisher’s exact test was used to compare the number of individuals (NSAID users vs nonusers) who had a focal amplification to the number of individuals who did not.

**Computational environment**

All computations were performed on a Linux server running Ubuntu 14.04.3 with the bulk of the analysis done in Matlab R2016b (9.1.0.441655), with SCA calls (excluding small HD calls) performed in Python 3.4.3, and deconstructSigs analysis performed in Python 3.5.1 with Pandas 0.19.1, Statsmodels 0.6.1, and Numpy 1.11.2. ADTEx was run with Python 2.7 and R 3.2, with deconstructSigs run on R 3.3. Mixed effect model analyses were carried out using SAS 9.4.

**References**

1. Levine DS, Blount PL, Rudolph RE, Reid BJ: Safety of a systematic endoscopic biopsy protocol in patients with Barrett's esophagus**.** *Am J Gastroenterol* 2000, 95**:**1152-1157.

2. Vaughan TL, Kristal AR, Blount PL, Levine DS, Galipeau PC, Prevo LJ, Sanchez CA, Rabinovitch PS, Reid BJ: Nonsteroidal anti-inflammatory drug use, body mass index, and anthropometry in relation to genetic and flow cytometric abnormalities in Barrett's esophagus**.** *Cancer Epidemiol Biomarkers Prev* 2002, 11**:**745-752.

3. Ogino S, Chan AT, Fuchs CS, Giovannucci E: Molecular pathological epidemiology of colorectal neoplasia: an emerging transdisciplinary and interdisciplinary field**.** *Gut* 2011, 60**:**397-411.

4. Vaughan TL, Kristal AR, Blount PL, Levine DS, Galipeau PC, Prevo LJ, Sanchez CA, PS R, BJ R: NSAID use, BMI, and anthropometry in relation to genetic and cell cycle abnormalities in Barrett's Esophagus**.** *Cancer Epidemiology, Biomarkers & Prevention* 2002, 11**:**745-752.

5. Vaughan TL, Dong LM, Blount PL, Ayub K, Odze RD, Sanchez CA, Rabinovitch PS, Reid BJ: Non-steroidal anti-inflammatory drugs and risk of neoplastic progression in Barrett's oesophagus: a prospective study**.** *Lancet Oncol* 2005, 6**:**945-952.

6. Farrow DC, Vaughan TL, Hansten PD, Stanford JL, Risch HA, Gammon MD, Chow WH, Dubrow R, Ahsan H, Mayne ST, et al: Use of aspirin and other nonsteroidal anti-inflammatory drugs and risk of esophageal and gastric cancer**.** *Cancer Epidemiol Biomarkers Prev* 1998, 7**:**97-102.

7. Vaughan TL, Farrow DC, Hansten PD, Chow WH, Gammon MD, Risch HA, Stanford JL, Schoenberg JB, Mayne ST, Rotterdam H, et al: Risk of esophageal and gastric adenocarcinomas in relation to use of calcium channel blockers, asthma drugs, and other medications that promote gastroesophageal reflux**.** *Cancer Epidemiol Biomarkers Prev* 1998, 7**:**749-756.

8. Li X, Galipeau PC, Paulson TG, Sanchez CA, Arnaudo J, Liu K, Sather CL, Kostadinov RL, Odze RD, Kuhner MK, et al: Temporal and spatial evolution of somatic chromosomal alterations: a case-cohort study of Barrett's esophagus**.** *Cancer Prev Res (Phila)* 2014, 7**:**114-127.

9. Li H, Durbin R: Fast and accurate short read alignment with Burrows-Wheeler transform**.** *Bioinformatics* 2009, 25**:**1754-1760.

10. Li H, Handsaker B, Wysoker A, Fennell T, Ruan J, Homer N, Marth G, Abecasis G, Durbin R, Genome Project Data Processing S: The Sequence Alignment/Map format and SAMtools**.** *Bioinformatics* 2009, 25**:**2078-2079.

11. DePristo MA, Banks E, Poplin R, Garimella KV, Maguire JR, Hartl C, Philippakis AA, del Angel G, Rivas MA, Hanna M, et al: A framework for variation discovery and genotyping using next-generation DNA sequencing data**.** *Nat Genet* 2011, 43**:**491-498.

12. Van der Auwera GA, Carneiro MO, Hartl C, Poplin R, Del Angel G, Levy-Moonshine A, Jordan T, Shakir K, Roazen D, Thibault J, et al: From FastQ data to high confidence variant calls: the Genome Analysis Toolkit best practices pipeline**.** *Curr Protoc Bioinformatics* 2013, 43**:**11 10 11-33.

13. Cibulskis K, Lawrence MS, Carter SL, Sivachenko A, Jaffe D, Sougnez C, Gabriel S, Meyerson M, Lander ES, Getz G: Sensitive detection of somatic point mutations in impure and heterogeneous cancer samples**.** *Nat Biotechnol* 2013, 31**:**213-219.

14. Saunders CT, Wong WS, Swamy S, Becq J, Murray LJ, Cheetham RK: Strelka: accurate somatic small-variant calling from sequenced tumor-normal sample pairs**.** *Bioinformatics* 2012, 28**:**1811-1817.

15. Spurrier JD: On the null distribution of the Kruskal-Wallis statistic**.** *Journal of Nonparametric Statistics* 2003, 15**:**685-691.

16. Pruitt KD, Tatusova T, Maglott DR: NCBI Reference Sequence (RefSeq): a curated non-redundant sequence database of genomes, transcripts and proteins**.** *Nucleic Acids Res* 2005, 33**:**D501-504.

17. Karolchik D, Hinrichs AS, Furey TS, Roskin KM, Sugnet CW, Haussler D, Kent WJ: The UCSC Table Browser data retrieval tool**.** *Nucleic Acids Res* 2004, 32**:**D493-496.

18. Sprent P: *Applied Nonparametric Statistical Methods.* 2nd edn: Chapman & Hall; 1989.

19. Littell RC, Milliken GA, Stroup WW, Wolfinger RD, Schabenberger O: *SAS for Mixed Models.* 2nd edn: SAS Institute; 2006.

20. Alexandrov LB, Nik-Zainal S, Wedge DC, Campbell PJ, Stratton MR: Deciphering signatures of mutational processes operative in human cancer**.** *Cell Rep* 2013, 3**:**246-259.

21. Nones K, Waddell N, Wayte N, Patch AM, Bailey P, Newell F, Holmes O, Fink JL, Quinn MC, Tang YH, et al: Genomic catastrophes frequently arise in esophageal adenocarcinoma and drive tumorigenesis**.** *Nat Commun* 2014, 5**:**5224.

22. Forbes SA, Beare D, Boutselakis H, Bamford S, Bindal N, Tate J, Cole CG, Ward S, Dawson E, Ponting L, et al: COSMIC: somatic cancer genetics at high-resolution**.** *Nucleic Acids Res* 2017, 45**:**D777-D783.

23. Rosenthal R, McGranahan N, Herrero J, Taylor BS, Swanton C: DeconstructSigs: delineating mutational processes in single tumors distinguishes DNA repair deficiencies and patterns of carcinoma evolution**.** *Genome Biol* 2016, 17**:**31.

24. Tuglus C, van der Laan MJ: Modified FDR controlling procedure for multi-stage analyses**.** *Stat Appl Genet Mol Biol* 2009, 8**:**Article 12.

25. Benjamini Y, Hochberg Y: Controlling the False Discovery Rate: A Practical and Powerful Approach to Multiple Testing**.** *J Roy Stat Soc, Ser B* 1995, 57**:**289-300.

26. Pratt D, Chen J, Welker D, Rivas R, Pillich R, Rynkov V, Ono K, Miello C, Hicks L, Szalma S, et al: NDEx, the Network Data Exchange**.** *Cell Syst* 2015, 1**:**302-305.

27. Schaefer CF, Anthony K, Krupa S, Buchoff J, Day M, Hannay T, Buetow KH: PID: the Pathway Interaction Database**.** *Nucleic Acids Res* 2009, 37**:**D674-679.

28. Fabregat A, Sidiropoulos K, Garapati P, Gillespie M, Hausmann K, Haw R, Jassal B, Jupe S, Korninger F, McKay S, et al: The Reactome pathway Knowledgebase**.** *Nucleic Acids Res* 2016, 44**:**D481-487.

29. Milacic M, Haw R, Rothfels K, Wu G, Croft D, Hermjakob H, D'Eustachio P, Stein L: Annotating cancer variants and anti-cancer therapeutics in reactome**.** *Cancers (Basel)* 2012, 4**:**1180-1211.

30. Yates B, Braschi B, Gray KA, Seal RL, Tweedie S, Bruford EA: Genenames.org: the HGNC and VGNC resources in 2017**.** *Nucleic Acids Res* 2017, 45**:**D619-D625.

31. Gray KA, Yates B, Seal RL, Wright MW, Bruford EA: Genenames.org: the HGNC resources in 2015**.** *Nucleic Acids Res* 2015, 43**:**D1079-1085.

32. Kang YJ, Mbonye UR, DeLong CJ, Wada M, Smith WL: Regulation of intracellular cyclooxygenase levels by gene transcription and protein degradation**.** *Prog Lipid Res* 2007, 46**:**108-125.

33. Peiffer DA, Le JM, Steemers FJ, Chang W, Jenniges T, Garcia F, Haden K, Li J, Shaw CA, Belmont J, et al: High-resolution genomic profiling of chromosomal aberrations using Infinium whole-genome genotyping**.** *Genome Res* 2006, 16**:**1136-1148.

34. Amarasinghe KC, Li J, Hunter SM, Ryland GL, Cowin PA, Campbell IG, Halgamuge SK: Inferring copy number and genotype in tumour exome data**.** *BMC Genomics* 2014, 15**:**732.

35. Littell RC, Freund RJ, Spector PC: *SAS system for linear models.* 3rd edn: SAS Institute; 1991.
