# Supplementary material for: NSAID use and somatic exomic mutations in Barrett’s esophagus
Source: Genome Med. 2018 Feb 27;10:17. doi: 10.1186/s13073-018-0520-y (PMC5830331; doi:10.1186/s13073-018-0520-y)
Supplement: Supplementary file 3 — Supplemental Figures: Additional file 3 includes Supplemental Figures S1-S7. (PDF 1067 kb) [file 13073_2018_520_MOESM3_ESM.pdf]

Supplemental Figure S1. Study design

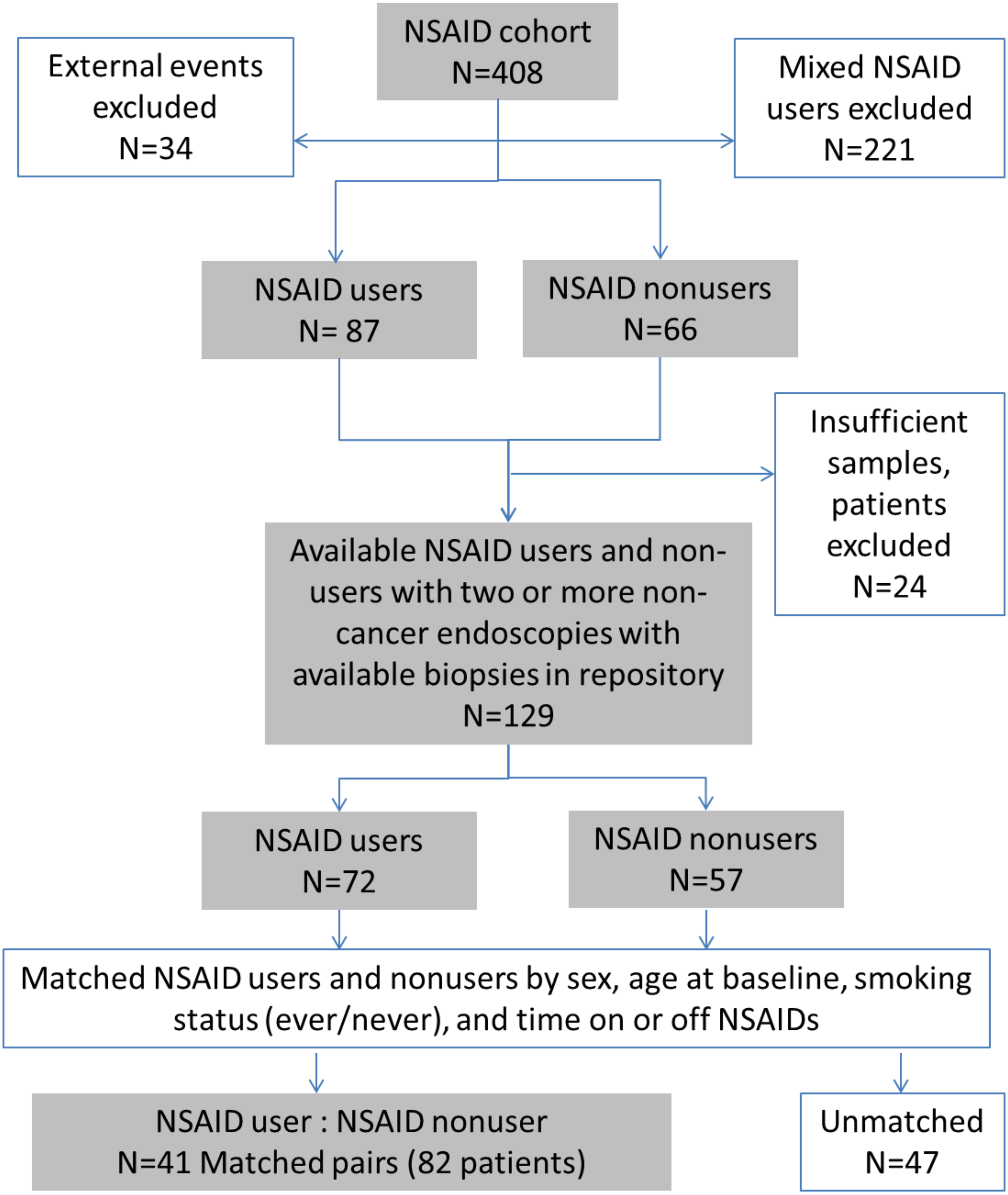

Figure S1. Flow chart for cross-sectional study design.

Supplemental Figure S2. Probability distribution of mutations per NSAID use/smoking status group

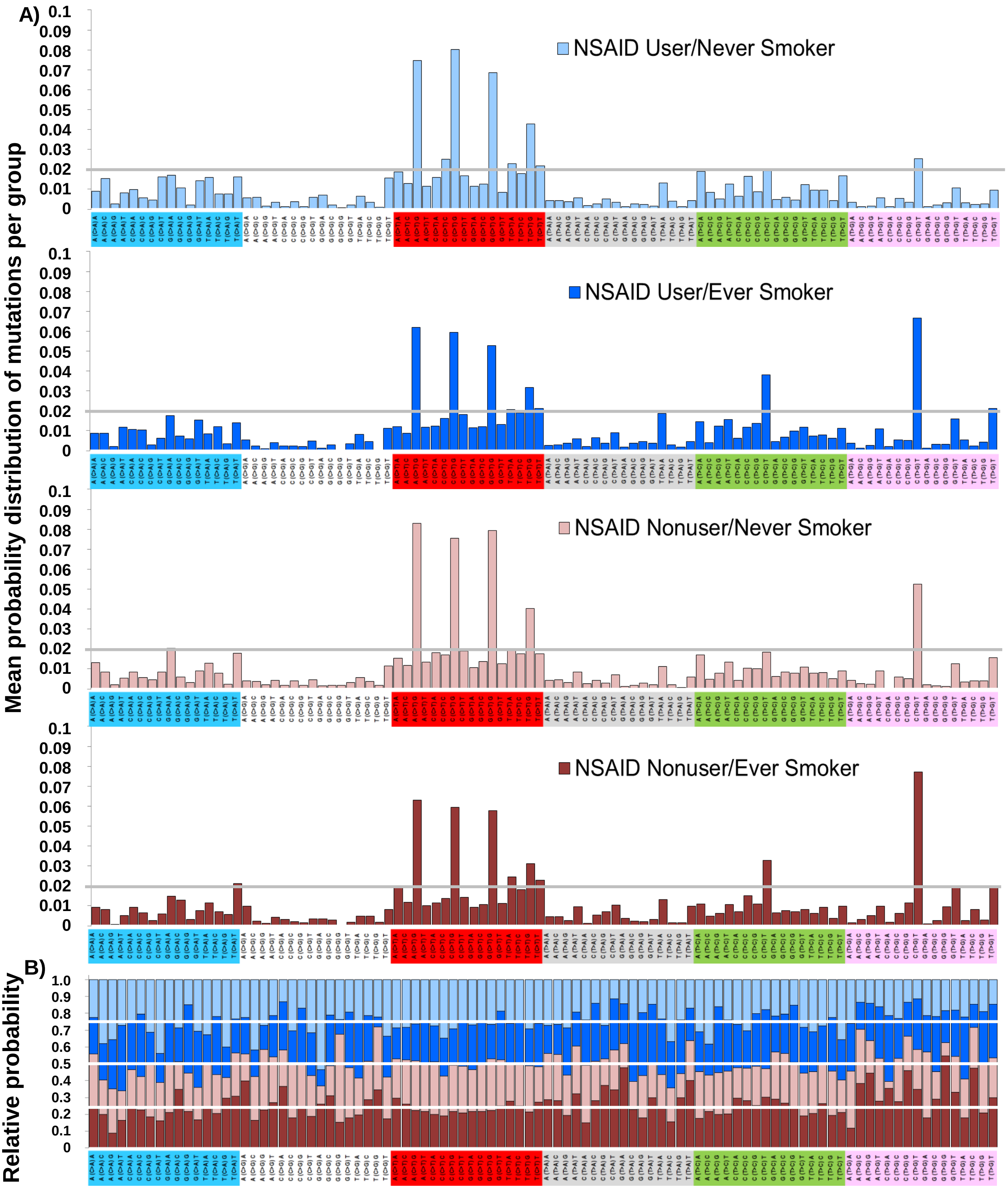

Figure S2. **A)** The number of mutations of each of the 96 mutation types per patient were normalized by the patient's total number of mutations to generate a probability distribution of tri-nucleotide mutation likelihood for that patient. These were then averaged across patients of each group by taking the mean probability of mutation for each mutation type, across the patients within the group. **B)** Mean probability from each group scaled by the sum of probabilities across groups per mutation type.

Supplemental Figure S3. *de novo* mutation signature analysis in all patients

Cosmic Mutational Signatures in Cancer

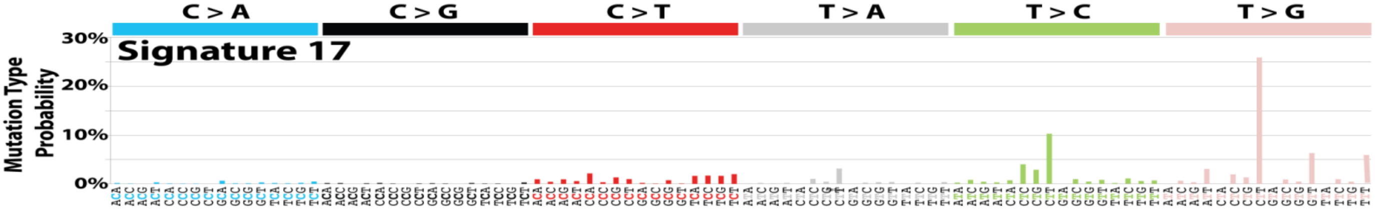

BE patients N=82

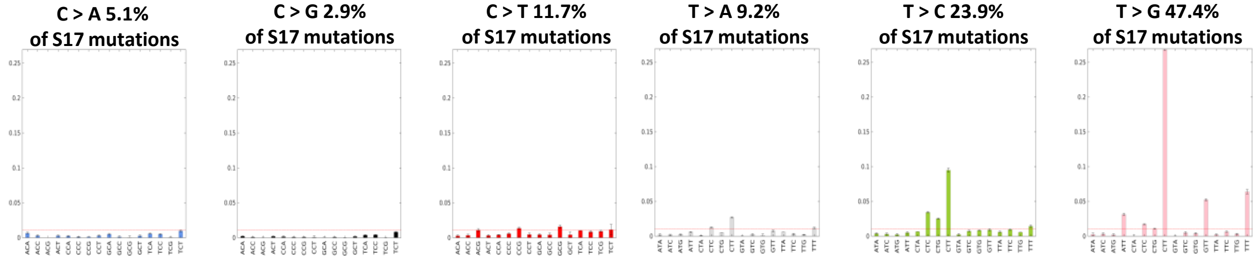

Cosmic Mutational Signatures in Cancer

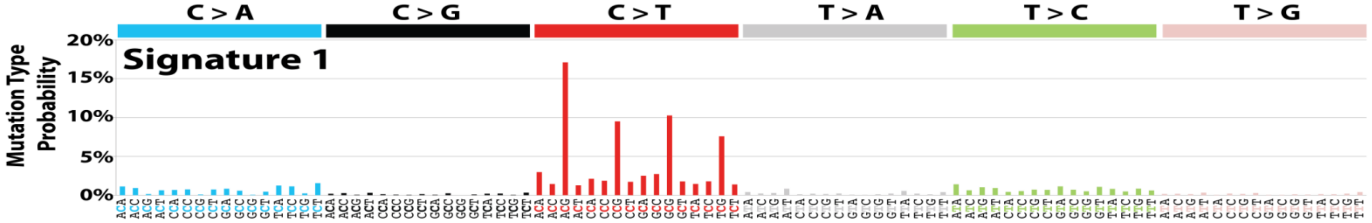

BE patients N=82

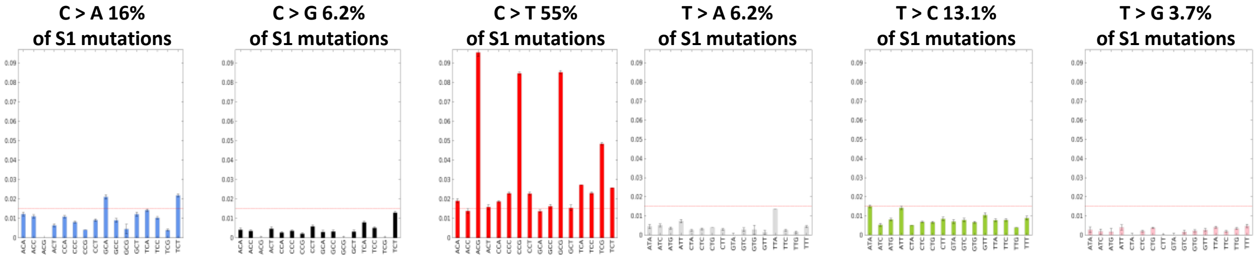

Figure S3. Two high-confidence mutational signatures obtained from 82 BE patients are shown below the canonical Cosmic Mutational Signatures in Cancer (http://cancer.sanger.ac.uk/cosmic/signatures) Signatures 17 and Signature 1. See Additional file 1 for detailed methods.

Supplemental Figure S4. SCA in users and nonusers

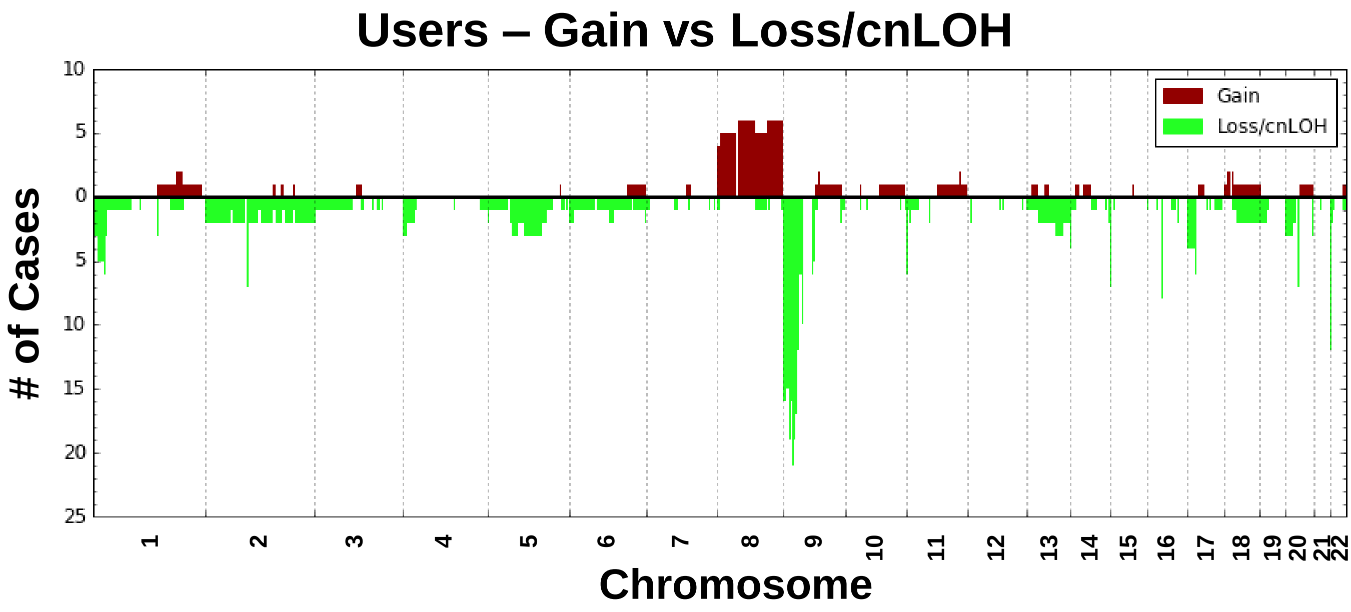

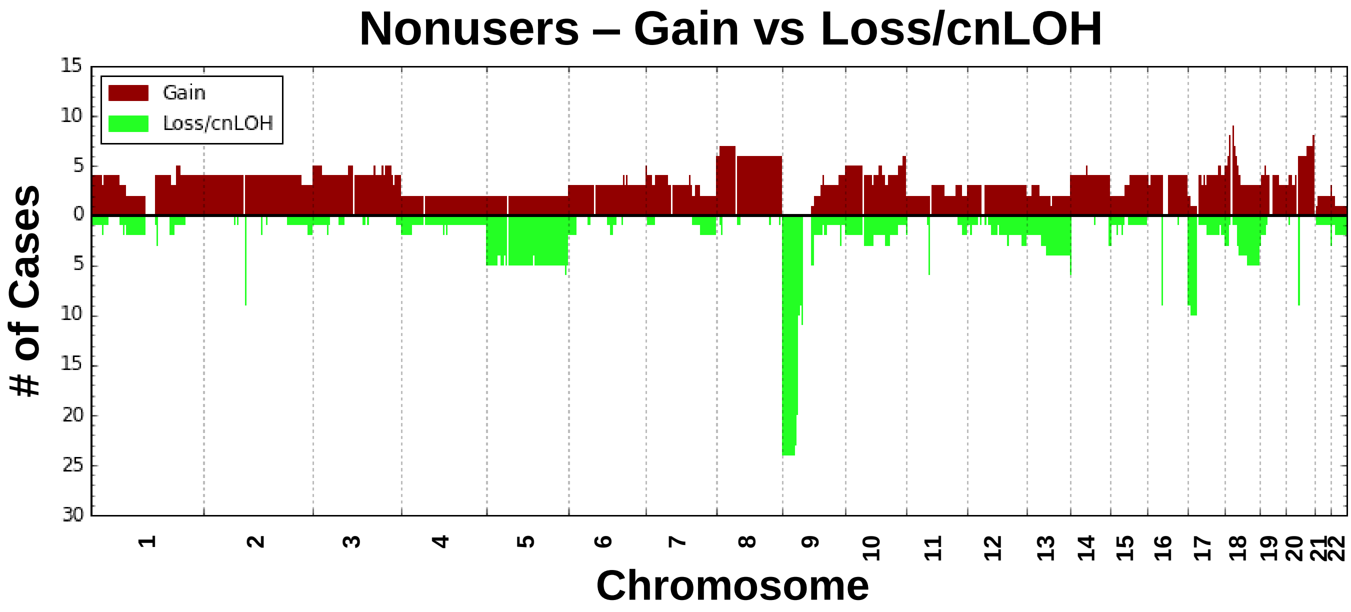

Figure S4. Red (above zero) is the number of patients with allele specific copy gain, balanced gain, or high gain. Green (below zero) is any homozygous deletion, loss, or cnLOH. The top plot is for NSAID users and the bottom plot is for NSAID nonusers. X axis is chromosome location. The Y-axis is number of samples with any event (41 user and 41 nonusers samples). Note that homozygous deletions are too small to be represented in this plot.

Supplemental Figure S5. SCA in individual samples

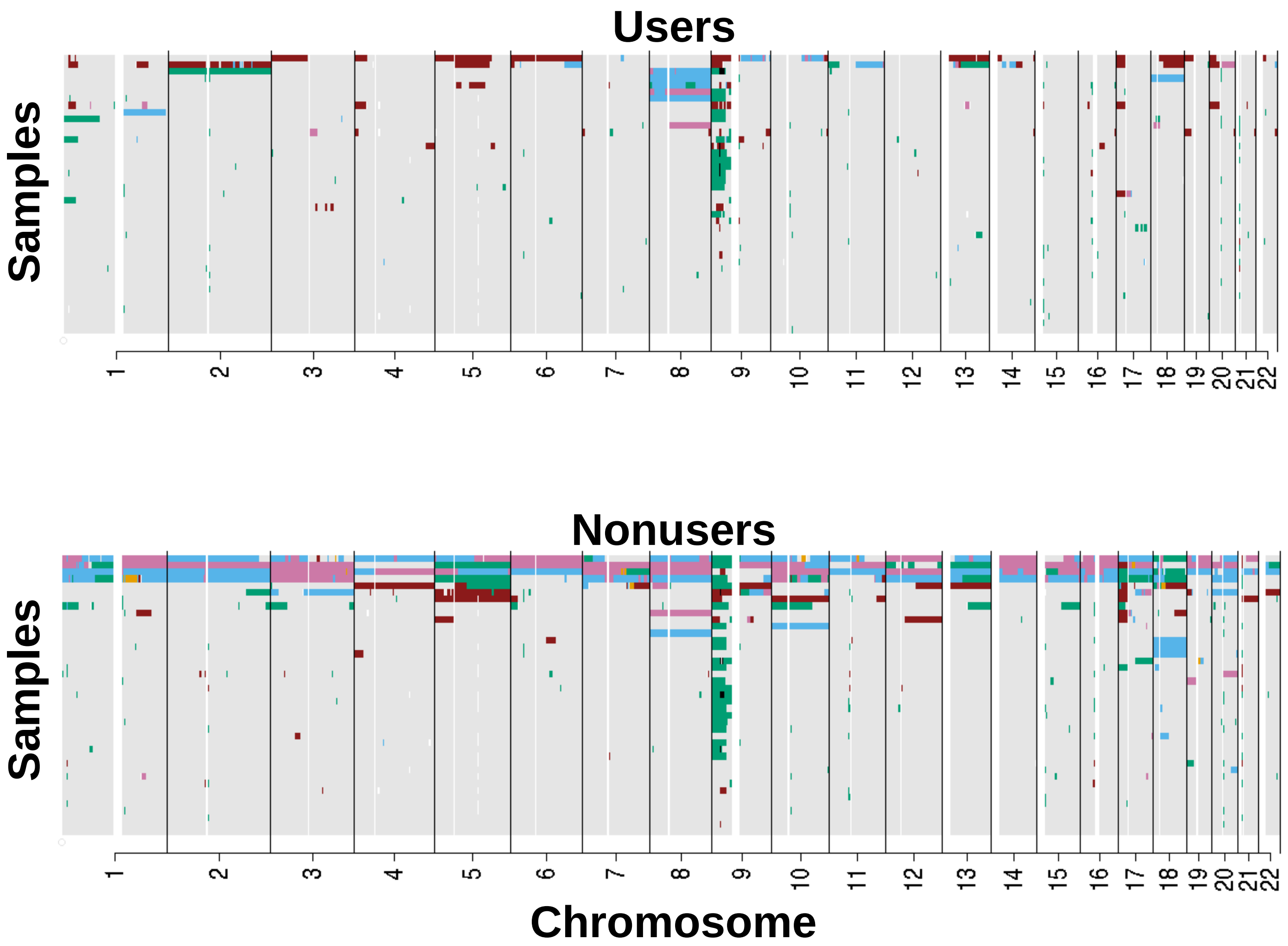

Figure S5. SCA in each sample is displayed per row, ordered by highest total SCA at the top. X axis is chromosome location. The Y-axis is individual samples (41 user samples and 41 nonuser samples). The white spaces in the middle of each chromosome represent centromeres; the few non-centromere white spaces represent regions with insufficient data to make a call.

Supplemental Figure S6. Test for BE:NL matching based on proportion of matched genotypes.

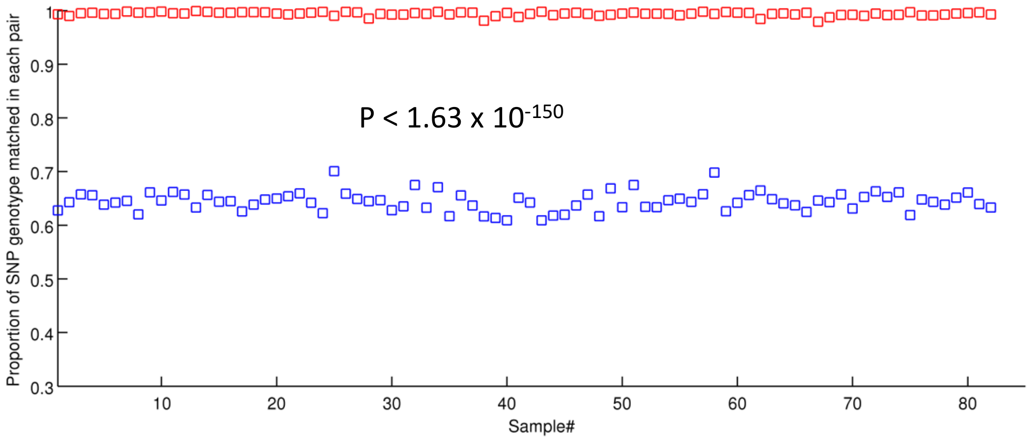

Figure S6. Normal blood control samples were compared to the BE sample from the same patient to verify the pairs were from the same individual based upon the proportion of matched SNPs for each pair. Red boxes indicate the proportion of matching SNPs when samples from the same individual were compared. Blue boxes indicate the proportion of matching SNPs when the normal was compared to a random, non-matching BE sample.

Supplemental Figure S7. Depth of sequence coverage comparison

	NSAID Nonusers		NSAID Users	
	**# Reads (billion)**	**Mean DOC**	**# Reads (billion)**	**Mean DOC**
**Normals (N=41)**				
average	8.14	84.81	8.21	85.5
min	5.98	62.29	5.26	54.77
max	10.64	110.78	15.15	157.75
**BE (N=41)**				
average	7.64	79.6	7.75	80.69
min	5.74	59.76	3.76	39.21
max	10	104.11	11.41	118.81

Figure S7. Depth of coverage target in exome sequence was average coverage of 80X. Details provided in Supplemental Table S20.
